# Supplementary material for: A Phase 2a, Randomized, Double-Blind, Placebo-Controlled Study to Assess the Efficacy and Safety of ALS-L1023 in Non-Alcoholic Fatty Liver Disease
Source: Pharmaceuticals (Basel). 2023 Apr 20;16(4):623. doi: 10.3390/ph16040623 (PMC10142612; doi:10.3390/ph16040623)

Supplementary Online Content

Table of contents

Supplementary Methods: Inclusion Criteria, Exclusion Criteria

Table S1. Changes in serum biomarkers from baseline to week 24 in the PPS
Table S2. Changes in serum biomarkers from baseline to week 8 and week 16 in the FAS
Table S3. Changes in serum biomarkers from baseline to week 8 and week 16 in the PPS
Table S4. Significantly changed miRNAs from ALS-L1023 treated patients
Table S5. 6 miRNAs and their putative target transcripts
Table S6. KEGG analysis using 499 putative target transcripts

Figure S1. Summary of study design
Figure S2. MRI-PDFF change in the PPS. MRI-PDFF results relative mean fat reduction percent from baseline (A), and absolute amount of fat reduction (B) as determined by MRI-PDFF at week 24
Figure S3. Change of liver biochemistry in the PPS. Time course of liver enzymes ALT (A), and AST (B). Time course of total cholesterol level (C).

Supplementary Methods

Inclusion Criteria

1) Subject is a male or female between 19 to 75 years of age inclusive
2) Subjects diagnosed with NAFLD (Nonalcoholic fatty liver disease) with findings of liver fat deposition by imaging tests such as abdominal ultrasonography and MRI
3) Subjects with liver fat content ≥8% as measured by MRI-PDFF within 90 days before screening and with liver fibrosis ≥2.5 kPa as measured by MRE
4) Patients who voluntarily decide to participate and agree in writing to abide by the precautions after hearing and fully understanding the detailed explanation of this clinical trial

Exclusion Criteria

1) Subjects who consumed significant alcohol for more than 3 consecutive months within 2 years before screening (significant alcohol intake: >210 g/week for men, >140 g/week for women)
2) Subjects with a history or presence of the following liver diseases
   - Current history of acute and chronic viral hepatitis (including HBsAg and HCV Ab positive in screening for hepatitis B and C)
   - Decompensated liver cirrhosis or liver cancer
   - Autoimmune liver disease (e.g., autoimmune hepatitis, primary biliary cirrhosis, primary sclerosing cholangitis, immunoglobulin G4-related cholangitis, etc.)
   - Alcoholic liver disease or drug-induced liver disease
   - Hereditary liver disease (e.g. Wilson's disease, alpha-1 antitrypsin deficiency, hemochromatosis, etc.)
3) Subjects with a history of uncontrolled hypertension (≥180/110 mmHg), severe heart disease (e.g. heart failure, unstable angina, myocardial infarction, arrhythmia requiring treatment, etc.), or severe cerebrovascular disease (e.g. cerebral infarction, cerebral hemorrhage, etc.). However, a subject could participate if an investigator judged that a subject could participate in this clinical trial because the subject was clinically stable.
4) Subjects with a history of malignant tumors. However, participation is possible if there is no recurrence for more than 5 years after being cured.
5) Subjects who have undergone a surgical operation (e.g. bariatric surgery) for weight loss within 5 years before screening
6) Patients with type 1 diabetes or uncontrolled diabetes mellitus (HbA1C >9.0% at screening)
7) Subjects with a clinically significant history of kidney disease (Serum creatinine >Upper limits of normal (ULN) at screening)
8) Uncontrolled Hypothyroidism (TSH ≥2X Upper limits of normal (ULN) at screening) or uncontrolled thyroid dysfunction (if not controlled for more than 6 months before screening

according to the investigator's judgment)

9) If ALT or AST is more than 5 times the upper limit of normal at the before-screening test

10) Subjects who have continuously administered the following drugs for more than 2 weeks within 8 weeks before screening
   - Systemic Glucocorticoids (inhalation, topical steroids (e.g. nasal spray, eye drops, intra-articular administration, etc.) administration are allowed)
   - Ibuprofen, Naproxen

11) Subjects who have administered the following drugs within 8 weeks before screening
   - Polyunsaturated fatty acids (>2 g/day)
   - Immunomodulators (e.g. Azathioprine, 6-mercaptopurine, Methotrexate, Cyclosporin, Anti-TNF-α, Anti-integrin)
   - Irinotecan, Tamoxifen, 5-fluorouracil, Cisplatin, Asparaginase
   - Valproic acid
   - Amiodarone
   - Antiobesity drugs (e.g. Orlistat, Phentermine, Diethylpropion, Mazindol, Lorcaserin, Liraglutide, etc.)

12) Subjects who need to be administered the following drugs that are judged to affect the efficacy evaluation results during clinical trials. However, if the following conditions are satisfied, randomization is possible
   - UDCA (≥150 mg/day): Randomization possible after a washout period of 2 weeks or more
   - SAMe, Betaine, Milk thistle, Pentoxifylline: randomization possible after a 1-week washout period
   - Antihyperlipidemic drugs (e.g. Statins, Fibrates, Ezetimibe, etc.), Angiotensin Receptor Blocker (e.g. Losartan, etc.): randomization is possible if administered without changing usage or dosage for more than 12 weeks before screening
   - Diabetes drugs other than thiazolidinediones (e.g. Metformin, Sulfonylureas, Insulin, etc.), Thyroid Hormone formulations (e.g. Levothyroxine sodium, etc.): randomization is possible if administered at a stable dose for more than 12 weeks before screening
   - Vitamin E (>400 IU/day), Thiazolidinediones (Pioglitazone, Rosiglitazone): randomization is possible if administered without changing usage or dosage for more than 6 months before screening
   - Drugs that may affect the efficacy evaluation results according to the judgment of other investigators

13) Subjects with hypersensitivity to an investigational drug or components of an investigational drug such as Melissa, microcrystalline cellulose, hydroxypropyl cellulose, etc.

14) Subjects who cannot tolerate the noise in the examination equipment or isolation environment in the MRI room, or who are contraindicated in MRI examination (e.g. cardiac pacemakers, metallic vascular clips, those who wear iron implants or devices in the body, those who insert prostheses into the eyes or ears, and others who have foreign substances in their body that can be affected by magnetic fields)

15) Women of childbearing potential who do not consent to contraception by a medically accepted method of contraception during the clinical trial period (hormonal therapy, IUD (intrauterine device) or IUS (Intrauterine system), tubal ligation, double blocking method (combined use of blocking methods such

as male condoms, female condoms, cervical caps, contraceptive diaphragms, and contraceptive sponges, etc.)

16) Pregnant or lactating women
17) Subjects who received other clinical investigational drugs within 30 days before screening
18) If the investigator judges that the subject is unsuitable for this clinical trial by other reasons

Table S1. Changes in serum biomarkers from baseline to week 24 in the PPS

	ALS-L1023 1800mg			ALS-L1023 1200mg			Placebo			p^2	p^3
**Value**	**CFB**	**% CFB**	p^1	**CFB**	**% CFB**	p^1	**CFB**	**% CFB**	p^1		
ALT, U/L	-12 (-19, 3)	-13.5 (-30.2, 6.7)	0.07	-17 (-40, 0)	-36.0 (-47.8, 0)	0.10	1 (-7, 22)	0.7 (-15.4, 44.0)	0.45	0.04	0.03
AST, IU/L	-1 (-12, 9)	-1.5 (-21.7, 15.9)	0.47	-11 (-29, 4)	-21.0 (-41.7, 10.5)	0.15	0 (-6, 22)	0 (-16.1, 55.2)	0.57	0.37	0.15
GGT, IU/L	-3 (-12, 8)	-8.1 (-17.4, 17.2)	0.37	-3 (-37, 7)	-7.69 (-44.3, 11.9)	0.09	1 (-3, 6)	2.2 (-8.1, 23.1)	0.20	0.29	0.14
ALP, IU/L	1 (-8, 5)	0.6 (-8.9, 6.9)	0.76	-1 (-11, 5)	-1.06 (-14.0, 6.3)	0.45	-3 (-21, 7)	-3.6 (-18.8, 14.1)	0.13	0.29	0.35
Total bilirubin, mg/dL	-0.1 (-0.1, 0.3)	-8.0 (-19.0, 26.0)	0.81	0.1 (-0.1, 0.3)	15.25 (-13.6, 33.6)	0.07	0.2 (-0.3, 0.4)	17.2 (-21.4, 60.9)	0.32	0.33	0.77
Triglycerides, mg/dL	-32 (-52, 6)	-12.5 (-27.7, 29.9)	0.23	-7 (-44, 15)	-4.4 (-28.4, 11.4)	0.35	-34 (-70, -5)	-18.0 (-36.4, -2.7)	<0.01	0.40	0.14
TC, mg/dL	4 (-15,12)	1.6 (-8.9, 7.6)	0.83	-10 (-19, -4)	-5.8 (-12.9,-1.5)	<0.01	10 (-12,16)	5.4 (-6.3, 7.6)	0.33	0.40	<0.01
HDL-C, mg/dL	1 (-5, 4)	1.1 (-9.4, 8.6)	0.90	-1 (-7, 5)	-2.7 (-12.6, 11.5)	0.70	1 (-2, 7)	2.0 (-3.3, 14.9)	0.31	0.39	0.36
LDL-C, mg/dL	-3 (-13, 9)	-2.1 (-8.2, 9.5)	0.47	-7 (-23, 6)	-9.21 (-21.5, 5.7)	0.08	6 (0, 19)	6.1 (0, 12.2)	0.10	0.09	0.02
Pro-C3	-0 (-0.6, 1.3)	-1.0 (-6.2, 14.5)	0.77	-0.3 (-1.0, 1.2)	-3.7 (-12.6, 18.6)	0.78	-0.2 (-0.7, 1.1)	-2.0 (-10.9, 17.6)	0.96	0.81	0.83
CK-18	29.7 (-83.7, 100.2)	13.7 (-34.0, 47.0)	0.67	-61.7 (-192.4, 40.4)	-18.3 (-56.2, 30.7)	0.21	-3.7 (-128.6, 206.7)	-10.5 (-23.2, 101.1)	0.94	0.90	0.39
HOMA-IR	0.12 (-1.5, 1.9)	4.1 (-25.5, 41.9)	0.98	-0.16 (-4.0, 1.0)	-6.03 (-57.2, 45.2)	0.16	0.15 (-1.6, 1.3)	4.99 (-32.7, 36.1)	0.96	0.93	0.58
Leptin	-0.7 (-5.8, 3.4)	-1.3 (-23.8,15.9)	0.77	0.2 (-5.8, 4.0)	2.5 (-14.6, 26.6)	0.85	-0.8 (-3.2, 1.4)	-5.0 (-16.8, 10.2)	0.22	0.81	0.73
Ghrelin	15.7 (0.0, 30.6)	30.8 (0, 152.1)	0.01	9.9 (-3.0, 23.3)	38.3 (-12.7, 76.4)	0.06	5.1 (0, 24.3)	10.0 (0.0, 90.5)	0.09	0.42	0.85
Adiponectin	325 (-824, 1011)	8.5 (-21.6, 13.6)	0.50	-587 (-2940, 982)	-6.9 (-26.2, 15.7)	0.52	491 (-1489, 728)	4.4 (-24.1, 13.7)	0.93	0.65	0.85
NFS	-0.1 (-0.3, 0.2)	12.4 (-19.9, 39.8)	0.27	0.3 (-0.3, 0.4)	-13.6 (-25.7, 14.4)	0.20	-0.03 (-0.4, 0.4)	0.9 (-23.2, 35.4)	0.95	0.45	0.35
Visceral Fat mass	-18.8 (-49.1, 17.9)	-8.9 (-30.2, 9.8)	0.16	-7.7 (-29.0, 21.0)	-11.4 (-17.8, 19.2)	0.95	-5.3 (-38.4, 25.4)	-4.9 (-30.9, 12.3)	0.61	0.52	0.49

Abbreviation: ALP, alkaline phosphatase; ALT, alanine aminotransferase; AST, aspartate aminotransferase; CFB: Change from baseline; GGT, gamma-glutamyl transferase; HDL, high-density lipoprotein; LDL, low-density lipoprotein; NFS, NAFLD fibrosis score; TC, total cholesterol
Values are median (interquartile range), CFB: Change from baseline
[1]CFB in the group was analyzed by Paired t-test or Wilcoxon signed rank test
[2]Comparison of CFBs in ALS-L1023 1800mg and Placebo groups by Two-sample t-test or Wilcoxon rank sum test
[3]Comparison of CFBs in ALS-L1023 1200mg and Placebo groups by Two-sample t-test or Wilcoxon rank sum test

Table S2. Changes in serum biomarkers from baseline to week 8 and week 16 in the FAS

		ALS-L1023 1800mg			ALS-L1023 1200mg			Placebo			p[2]	p[3]
Value		CFB	% CFB	p[1]	CFB	% CFB	p[1]	CFB	% CFB	p[1]		
ALT, U/L	Week 8	0 (-6.5, 4.3)	0 (-11.3, 6.2)	0.64	-11 (-35.5, -2)	-18.9 (-27.8, -3.6)	0.01	2 (-4, 19)	2.9 (-8.7, 39.6)	0.22	0.24	0.01
	Week 16	-9.5 (-16, 2.5)	-11.2 (-29.1, 2.5)	0.09	-9 (-41.5, 1.5)	-23.1 (-42.6, 6.3)	0.01	12 (-4, 30.5)	21 (-14.2, 46.7)	0.17	0.04	0.01
AST, IU/L	Week 8	2 (-6, 6)	4 (-15, 9.5)	0.76	-4 (-23.5, 3)	-11.8 (-29.5, 5.3)	0.22	1 (-9, 11)	2.2 (-20.3, 28.6)	0.93	0.97	0.28
	Week 16	-3 (-14.5, 3)	-7.3 (-25.4, 9.4)	0.41	-5 (-23, 4)	-12.5 (-30.8, 7.9)	0.08	6 (-3.5, 19.5)	9.9 (-6.4, 56.9)	0.28	0.17	0.04
TC, mg/dL	Week 8	-2.5 (-16.3, 5.8)	-1.7 (-11.4, 3.8)	0.22	-8 (-27, 1)	-3.8 (-13.1, 0.5)	0.01	-1 (-10, 14.5)	-0.7 (-7.2, 8.4)	0.97	0.29	0.05
	Week 16	-3 (-14, 2)	-1.7 (-9.3, 1.5)	0.16	-4 (-24, 0.5)	-2.9 (-12.8, 0.4)	0.03	6 (-8.5, 13)	3.8 (-5.6, 7.9)	0.51	0.13	0.04

Abbreviation: ALT, alanine aminotransferase; AST, aspartate aminotransferase; CFB: Change from baseline; TC, total cholesterol
Values are presented in median (interquartile range).
[1]CFB in the group was analyzed by Paired t-test or Wilcoxon signed rank test
[2]Comparison of CFBs in ALS-L1023 1800mg and Placebo groups by Two-sample t-test or Wilcoxon rank sum test
[3]Comparison of CFBs in ALS-L1023 1200mg and Placebo groups by Two-sample t-test or Wilcoxon rank sum test

Table S3. Changes in serum biomarkers from baseline to week 8 and week 16 in the PPS

Value		ALS-L1023 1800mg			ALS-L1023 1200mg			Placebo			p[2]	p[3]
		CFB	% CFB	p[1]	CFB	% CFB	p[1]	CFB	% CFB	p[1]		
ALT, U/L	Week 8	0 (-6.5, 4.3)	0 (-11.3, 6.2)	0.64	-11 (-35.5, -3)	-18.9 (-28.3, -4.8)	<0.01	2 (-3, 19)	2.9 (-6.3, 38)	0.22	0.2	<0.01
	Week 16	-9.5 (-16, 2.5)	-11.2 (-29.1, 2.5)	0.09	-14 (-41.5, -5.5)	-25 (-43, -10.5)	0.013	12 (-3, 30)	21 (-11.5, 41.4)	0.054	<0.01	<0.01
AST, IU/L	Week 8	2 (-6, 6)	4 (-15, 9.5)	0.76	-4 (-19.5, 1.5)	-11.8 (-32.7, 3.4)	0.1	1 (-7, 6)	2.2 (-17.9, 20.8)	0.85	0.97	0.19
	Week 16	-3 (-14.5, 3)	-7.3 (-25.4, 9.4)	0.41	-9 (-24.5, 2)	-23.7 (-32.2, 3.2)	0.07	6 (-2, 18)	9.9 (-6.3, 52.4)	0.3	0.18	0.04
TC, mg/dL	Week 8	-2.5 (-16.3, 5.8)	-1.7 (-11.4, 3.8)	0.22	-16 (-29.5, -0.5)	-9.7 (-15.5, -0.5)	<0.01	-1 (-9, 15)	-0.7 (-6.1, 8.5)	0.95	0.30	0.02
	Week 16	-3 (-14, 2)	-1.7 (-9.3, 1.5)	0.16	-4 (-23, -0.5)	-2.9 (-12.7, -0.3)	<0.01	6 (-8, 14)	3.8 (-4.4, 8.8)	0.53	0.12	0.02

Abbreviation: ALT, alanine aminotransferase; AST, aspartate aminotransferase; CFB: Change from baseline; TC, total cholesterol
Values are presented in median (interquartile range).
[1]CFB in the group was analyzed by Paired t-test or Wilcoxon signed rank test
[2]Comparison of CFBs in ALS-L1023 1800mg and Placebo groups by Two-sample t-test or Wilcoxon rank sum test
[3]Comparison of CFBs in ALS-L1023 1200mg and Placebo groups by Two-sample t-test or Wilcoxon rank sum test

Table S4. Significantly changed miRNAs from ALS-L1023 treated patients.

A.

**1,800 mg ALS-L1023 treated group (V5 vs V2)**			
	**miRNAs**	**Fold change (end of treatment/base line)**	**p**
1	hsa-let-7e-5p	**-1.756**	**0.006**
2	hsa-let-7f-5p	**-1.549**	**0.019**
3	hsa-miR-29a-3p	**-1.536**	**0.025**
4	hsa-miR-132-3p	**-1.644**	**0.015**
5	hsa-miR-1184	**-1.699**	**0.035**
6	hsa-miR-3124-5p	**-1.561**	**0.006**
7	hsa-miR-4253	**-1.508**	**0.035**
8	hsa-miR-4286	**-1.622**	**0.015**
9	hsa-miR-3180	**-1.580**	**0.020**
10	hsa-miR-550b-3p	**-1.559**	**0.046**
11	hsa-miR-4462	**-1.500**	**0.009**
12	hsa-miR-4487	**-1.605**	**0.029**
13	hsa-miR-4743-5p	**-1.569**	**0.002**
14	hsa-miR-5010-5p	**-1.562**	**0.004**
15	hsa-miR-664b-3p	**-1.696**	**0.003**
16	hsa-miR-6803-3p	**-1.503**	**0.012**
17	hsa-miR-6781-5p	**-1.523**	**0.063**

B.

1,200 mg ALS-L1023 treated group (V5 vs V2)			
	**miRNAs**	**Fold change (end of treatment/base line)**	**p**
1	hsa-miR-361-3p	-1.518	0.019
2	hsa-miR-455-3p	-1.561	0.002
3	hsa-miR-1306-5p	-1.531	0.021
4	hsa-miR-1976	-1.502	0.025
5	hsa-miR-4253	-1.582	0.011
6	hsa-miR-378f	-1.555	0.003
7	hsa-miR-4743-5p	-1.575	0.002
8	hsa-miR-6846-5p	-1.583	0.001
9	hsa-miR-8063	-1.547	0.024
10	hsa-miR-371b-5p	-1.641	0.010
11	hsa-miR-6781-5p	-1.572	0.006
12	hsa-miR-6784-5p	-1.509	0.007
13	hsa-miR-3188	-1.795	0.007

C.

placebo group (V5 vs V2)			
	miRNAs	**Fold change (end of treatment/base line)**	**p**
1	hsa-miR-28-3p	**1.550**	**0.038**
2	hsa-miR-139-5p	**1.609**	**0.034**
3	hsa-miR-636	**1.515**	**0.033**
4	hsa-miR-1224-5p	**-1.576**	**0.025**
5	hsa-miR-3188	**-1.542**	**0.057**
6	hsa-miR-4322	**-1.599**	**0.032**
7	hsa-miR-4640-5p	**-1.610**	**0.018**
8	hsa-miR-371b-5p	**-1.648**	**0.077**
9	hsa-miR-5001-3p	**1.511**	**0.049**
10	hsa-miR-5572	**-1.542**	**0.037**
11	hsa-miR-6721-5p	**-1.650**	**0.009**
12	hsa-miR-6781-5p	**-1.793**	**0.005**
13	hsa-miR-6784-5p	**-1.663**	**0.010**
14	hsa-miR-6815-5p	**-1.824**	**0.011**
15	hsa-miR-6889-5p	**-1.566**	**0.019**
16	hsa-miR-8073	**-1.658**	**0.028**

Table S5. 6 miRNAs and their putative target transcripts.

Gene Symbol		p-value	FDR	Odd ratio	Number of interactions	microRNA 1	microRNA 2	microRNA 3	microRNA 4	microRNA 5
1	AFF2	0.002	0.271	0.045	2	hsa-let-7e-5p/hsa-let-7f-5p	hsa-miR-132-3p			
2	APC2	0.002	0.271	0.045	2	hsa-let-7e-5p/hsa-let-7f-5p	hsa-miR-1306-5p			
3	C19orf47	0.001	0.271	0.034	2	hsa-let-7e-5p/hsa-let-7f-5p	hsa-miR-132-3p			
4	COL15A1	0.001	0.271	0.034	2	hsa-let-7e-5p/hsa-let-7f-5p	hsa-miR-29a-3p			
5	COL4A2	0.002	0.271	0.045	2	hsa-let-7e-5p/hsa-let-7f-5p	hsa-miR-29a-3p			
6	COL4A5	0.002	0.271	0.045	2	hsa-let-7e-5p/hsa-let-7f-5p	hsa-miR-29a-3p			
7	COL5A2	0.002	0.271	0.045	2	hsa-let-7e-5p/hsa-let-7f-5p	hsa-miR-29a-3p			
8	CTPS1	0.002	0.271	0.045	2	hsa-let-7e-5p/hsa-let-7f-5p	hsa-miR-29a-3p			
9	DUSP9	0.001	0.271	0.034	2	hsa-let-7e-5p/hsa-let-7f-5p	hsa-miR-132-3p			
10	GPATCH2	0.002	0.271	0.045	2	hsa-let-7e-5p/hsa-let-7f-5p	hsa-miR-29a-3p			
11	KIAA1549	0.002	0.271	0.182	4	hsa-let-7e-5p/hsa-let-7f-5p	hsa-miR-1306-5p	hsa-miR-132-3p	hsa-miR-29a-3p	
12	LMLN	0.002	0.271	0.114	3	hsa-let-7e-5p/hsa-let-7f-5p	hsa-miR-132-3p	hsa-miR-29a-3p		
13	LRRC59	0.002	0.271	0.114	3	hsa-let-7e-5p/hsa-let-7f-5p	hsa-miR-1306-5p	hsa-miR-29a-3p		
14	LYSMD1	0.002	0.271	0.045	2	hsa-miR-1306-5p	hsa-miR-29a-3p			
15	NKIRAS2	0.002	0.271	0.098	3	hsa-let-7e-5p/hsa-let-7f-5p	hsa-miR-1306-5p	hsa-miR-29a-3p		
16	NPAS4	0.002	0.271	0.098	3	hsa-miR-1306-5p	hsa-miR-29a-3p	hsa-miR-378f		
17	OTUB2	0.001	0.271	0.076	3	hsa-let-7e-5p/hsa-let-7f-5p	hsa-miR-29a-3p	hsa-miR-378f		
18	PDGFB	0.002	0.271	0.045	2	hsa-let-7e-5p/hsa-let-7f-5p	hsa-miR-29a-3p			
19	POLE3	0.001	0.271	0.034	2	hsa-miR-132-3p	hsa-miR-29a-3p			

20	PQLC2	0.001	0.271	0.034	2	hsa-let-7e-5p/hsa-let-7f-5p	hsa-miR-1306-5p			
21	PTAFR	0.002	0.271	0.045	2	hsa-let-7e-5p/hsa-let-7f-5p	hsa-miR-1306-5p			
22	RAB15	0.002	0.271	0.098	3	hsa-let-7e-5p/hsa-let-7f-5p	hsa-miR-29a-3p	hsa-miR-378f		
23	RAB40C	0.002	0.271	0.045	2	hsa-let-7e-5p/hsa-let-7f-5p	hsa-miR-29a-3p			
24	SLC2A3	0.002	0.271	0.098	3	hsa-miR-1306-5p	hsa-miR-29a-3p	hsa-miR-378f		
25	STARD9	0.002	0.271	0.045	2	hsa-let-7e-5p/hsa-let-7f-5p	hsa-miR-29a-3p			
26	TRIB2	0.002	0.271	0.114	3	hsa-let-7e-5p/hsa-let-7f-5p	hsa-miR-132-3p	hsa-miR-29a-3p		
27	AGO1	0.004	0.277	0.341	5	hsa-let-7e-5p/hsa-let-7f-5p	hsa-miR-1306-5p	hsa-miR-132-3p	hsa-miR-29a-3p	hsa-miR-378f
28	ALPK3	0.021	0.277	0.125	2	hsa-let-7e-5p/hsa-let-7f-5p	hsa-miR-378f			
29	AMOT	0.008	0.277	0.167	3	hsa-let-7e-5p/hsa-let-7f-5p	hsa-miR-132-3p	hsa-miR-29a-3p		
30	ARL10	0.008	0.277	0.080	2	hsa-miR-1306-5p	hsa-miR-378f			
31	ARRDC4	0.008	0.277	0.080	2	hsa-let-7e-5p/hsa-let-7f-5p	hsa-miR-29a-3p			
32	ATG9A	0.011	0.277	0.091	2	hsa-miR-1306-5p	hsa-miR-29a-3p			
33	B3GNT7	0.008	0.277	0.080	2	hsa-let-7e-5p/hsa-let-7f-5p	hsa-miR-1306-5p			
34	BCL7A	0.011	0.277	0.189	3	hsa-let-7e-5p/hsa-let-7f-5p	hsa-miR-1306-5p	hsa-miR-29a-3p		
35	BSND	0.008	0.277	0.080	2	hsa-let-7e-5p/hsa-let-7f-5p	hsa-miR-29a-3p			
36	BTG2	0.008	0.277	0.167	3	hsa-let-7e-5p/hsa-let-7f-5p	hsa-miR-132-3p	hsa-miR-29a-3p		
37	C2orf88	0.017	0.277	0.114	2	hsa-let-7e-5p/hsa-let-7f-5p	hsa-miR-29a-3p			
38	CACFD1	0.006	0.277	0.068	2	hsa-let-7e-5p/hsa-let-7f-5p	hsa-miR-29a-3p			
39	CACNG4	0.008	0.277	0.080	2	hsa-let-7e-5p/hsa-let-7f-5p	hsa-miR-29a-3p			
40	CALU	0.008	0.277	0.167	3	hsa-let-7e-5p/hsa-let-7f-5p	hsa-miR-132-3p	hsa-miR-29a-3p		

41	CCSAP	0.014	0.277	0.102	2	hsa-let-7e-5p/hsa-let-7f-5p	hsa-miR-29a-3p			
42	CDC42BPA	0.007	0.277	0.159	3	hsa-miR-132-3p	hsa-miR-29a-3p	hsa-miR-378f		
43	CDKN1A	0.017	0.277	0.114	2	hsa-let-7e-5p/hsa-let-7f-5p	hsa-miR-132-3p			
44	CHD8	0.017	0.277	0.114	2	hsa-miR-1306-5p	hsa-miR-132-3p			
45	COL11A1	0.004	0.277	0.057	2	hsa-let-7e-5p/hsa-let-7f-5p	hsa-miR-29a-3p			
46	COL1A2	0.008	0.277	0.080	2	hsa-let-7e-5p/hsa-let-7f-5p	hsa-miR-29a-3p			
47	COL24A1	0.011	0.277	0.091	2	hsa-let-7e-5p/hsa-let-7f-5p	hsa-miR-29a-3p			
48	COL27A1	0.014	0.277	0.102	2	hsa-let-7e-5p/hsa-let-7f-5p	hsa-miR-29a-3p			
49	COL3A1	0.004	0.277	0.057	2	hsa-let-7e-5p/hsa-let-7f-5p	hsa-miR-29a-3p			
50	COL4A1	0.017	0.277	0.114	2	hsa-let-7e-5p/hsa-let-7f-5p	hsa-miR-29a-3p			
51	COL4A6	0.004	0.277	0.057	2	hsa-let-7e-5p/hsa-let-7f-5p	hsa-miR-29a-3p			
52	COL9A1	0.014	0.277	0.102	2	hsa-let-7e-5p/hsa-let-7f-5p	hsa-miR-29a-3p			
53	CUEDC1	0.004	0.277	0.057	2	hsa-miR-29a-3p	hsa-miR-378f			
54	DAB1	0.007	0.277	0.159	3	hsa-let-7e-5p/hsa-let-7f-5p	hsa-miR-132-3p	hsa-miR-29a-3p		
55	DIAPH1	0.021	0.277	0.125	2	hsa-miR-1306-5p	hsa-miR-132-3p			
56	DKK3	0.017	0.277	0.114	2	hsa-let-7e-5p/hsa-let-7f-5p	hsa-miR-132-3p			
57	DPYSL3	0.007	0.277	0.159	3	hsa-let-7e-5p/hsa-let-7f-5p	hsa-miR-132-3p	hsa-miR-29a-3p		
58	DSG3	0.004	0.277	0.057	2	hsa-let-7e-5p/hsa-let-7f-5p	hsa-miR-29a-3p			
59	DUSP22	0.004	0.277	0.057	2	hsa-let-7e-5p/hsa-let-7f-5p	hsa-miR-29a-3p			
60	EIF2S2	0.003	0.277	0.121	3	hsa-let-7e-5p/hsa-let-7f-5p	hsa-miR-1306-5p	hsa-miR-29a-3p		
61	ELOVL4	0.021	0.277	0.125	2	hsa-let-7e-5p/hsa-let-7f-5p	hsa-miR-29a-3p			

62	EMB	0.014	0.277	0.102	2	hsa-let-7e-5p/hsa-let-7f-5p	hsa-miR-29a-3p			
63	ERCC6	0.004	0.277	0.057	2	hsa-let-7e-5p/hsa-let-7f-5p	hsa-miR-29a-3p			
64	ETNK2	0.017	0.277	0.114	2	hsa-let-7e-5p/hsa-let-7f-5p	hsa-miR-1306-5p			
65	FAM105B	0.014	0.277	0.102	2	hsa-miR-132-3p	hsa-miR-29a-3p			
66	FAM160B2	0.021	0.277	0.125	2	hsa-let-7e-5p/hsa-let-7f-5p	hsa-miR-1306-5p			
67	FAM57B	0.008	0.277	0.080	2	hsa-miR-1306-5p	hsa-miR-29a-3p			
68	FASTK	0.017	0.277	0.114	2	hsa-let-7e-5p/hsa-let-7f-5p	hsa-miR-29a-3p			
69	FBXL20	0.014	0.277	0.205	3	hsa-miR-132-3p	hsa-miR-29a-3p	hsa-miR-378f		
70	FREM2	0.021	0.277	0.125	2	hsa-let-7e-5p/hsa-let-7f-5p	hsa-miR-29a-3p			
71	GAB2	0.004	0.277	0.129	3	hsa-let-7e-5p/hsa-let-7f-5p	hsa-miR-1306-5p	hsa-miR-29a-3p		
72	GLIPR2	0.021	0.277	0.125	2	hsa-miR-1306-5p	hsa-miR-378f			
73	GOLGA7	0.014	0.277	0.102	2	hsa-let-7e-5p/hsa-let-7f-5p	hsa-miR-29a-3p			
74	GRPEL2	0.017	0.277	0.114	2	hsa-let-7e-5p/hsa-let-7f-5p	hsa-miR-29a-3p			
75	HAS2	0.017	0.277	0.220	3	hsa-let-7e-5p/hsa-let-7f-5p	hsa-miR-132-3p	hsa-miR-29a-3p		
76	HBEGF	0.004	0.277	0.136	3	hsa-let-7e-5p/hsa-let-7f-5p	hsa-miR-132-3p	hsa-miR-29a-3p		
77	HIF3A	0.011	0.277	0.091	2	hsa-let-7e-5p/hsa-let-7f-5p	hsa-miR-29a-3p			
78	ICOS	0.006	0.277	0.068	2	hsa-let-7e-5p/hsa-let-7f-5p	hsa-miR-29a-3p			
79	IGSF1	0.008	0.277	0.080	2	hsa-let-7e-5p/hsa-let-7f-5p	hsa-miR-29a-3p			
80	IRGQ	0.011	0.277	0.091	2	hsa-let-7e-5p/hsa-let-7f-5p	hsa-miR-29a-3p			
81	KATNBL1	0.008	0.277	0.167	3	hsa-let-7e-5p/hsa-let-7f-5p	hsa-miR-1306-5p	hsa-miR-29a-3p		
82	KCNJ12	0.014	0.277	0.102	2	hsa-miR-132-3p	hsa-miR-29a-3p			

83	KCNV1	0.008	0.277	0.080	2	hsa-let-7e-5p/hsa-let-7f-5p	hsa-miR-29a-3p			
84	KIAA0895L	0.008	0.277	0.080	2	hsa-let-7e-5p/hsa-let-7f-5p	hsa-miR-29a-3p			
85	KIF2A	0.021	0.277	0.235	3	hsa-let-7e-5p/hsa-let-7f-5p	hsa-miR-132-3p	hsa-miR-378f		
86	LOXL4	0.008	0.277	0.080	2	hsa-let-7e-5p/hsa-let-7f-5p	hsa-miR-29a-3p			
87	LSM11	0.004	0.277	0.136	3	hsa-let-7e-5p/hsa-let-7f-5p	hsa-miR-132-3p	hsa-miR-29a-3p		
88	MED28	0.021	0.277	0.125	2	hsa-let-7e-5p/hsa-let-7f-5p	hsa-miR-29a-3p			
89	MEPCE	0.014	0.277	0.102	2	hsa-miR-1306-5p	hsa-miR-132-3p			
90	MIF4GD	0.008	0.277	0.080	2	hsa-miR-1306-5p	hsa-miR-29a-3p			
91	NUP155	0.008	0.277	0.080	2	hsa-let-7e-5p/hsa-let-7f-5p	hsa-miR-378f			
92	PAG1	0.019	0.277	0.227	3	hsa-let-7e-5p/hsa-let-7f-5p	hsa-miR-29a-3p	hsa-miR-378f		
93	PAIP2	0.021	0.277	0.125	2	hsa-miR-132-3p	hsa-miR-29a-3p			
94	PCGF3	0.017	0.277	0.220	3	hsa-let-7e-5p/hsa-let-7f-5p	hsa-miR-132-3p	hsa-miR-29a-3p		
95	PIAS4	0.021	0.277	0.125	2	hsa-let-7e-5p/hsa-let-7f-5p	hsa-miR-29a-3p			
96	PIK3IP1	0.014	0.277	0.102	2	hsa-let-7e-5p/hsa-let-7f-5p	hsa-miR-132-3p			
97	POC1B-GALNT4	0.006	0.277	0.152	3	hsa-let-7e-5p/hsa-let-7f-5p	hsa-miR-1306-5p	hsa-miR-132-3p		
98	PRICKLE2	0.016	0.277	0.212	3	hsa-miR-1306-5p	hsa-miR-132-3p	hsa-miR-29a-3p		
99	RASGRP1	0.008	0.277	0.080	2	hsa-let-7e-5p/hsa-let-7f-5p	hsa-miR-132-3p			
100	RB1	0.011	0.277	0.091	2	hsa-let-7e-5p/hsa-let-7f-5p	hsa-miR-132-3p			
101	RCSD1	0.006	0.277	0.068	2	hsa-let-7e-5p/hsa-let-7f-5p	hsa-miR-1306-5p			
102	RIOK3	0.014	0.277	0.102	2	hsa-let-7e-5p/hsa-let-7f-5p	hsa-miR-29a-3p			
103	RIT1	0.017	0.277	0.114	2	hsa-miR-29a-3p	hsa-miR-378f			

104	SALL3	0.017	0.277	0.114	2	hsa-let-7e-5p/hsa-let-7f-5p	hsa-miR-132-3p			
105	SAP30L	0.013	0.277	0.197	3	hsa-let-7e-5p/hsa-let-7f-5p	hsa-miR-132-3p	hsa-miR-29a-3p		
106	SARM1	0.011	0.277	0.091	2	hsa-miR-29a-3p	hsa-miR-378f			
107	SFT2D3	0.008	0.277	0.080	2	hsa-let-7e-5p/hsa-let-7f-5p	hsa-miR-378f			
108	SH3RF3	0.011	0.277	0.091	2	hsa-let-7e-5p/hsa-let-7f-5p	hsa-miR-29a-3p			
109	SIX5	0.021	0.277	0.125	2	hsa-miR-29a-3p	hsa-miR-378f			
110	SLC10A7	0.006	0.277	0.152	3	hsa-let-7e-5p/hsa-let-7f-5p	hsa-miR-132-3p	hsa-miR-29a-3p		
111	SLC16A14	0.017	0.277	0.114	2	hsa-let-7e-5p/hsa-let-7f-5p	hsa-miR-29a-3p			
112	SLC2A14	0.021	0.277	0.125	2	hsa-miR-29a-3p	hsa-miR-378f			
113	SLC31A1	0.006	0.277	0.152	3	hsa-let-7e-5p/hsa-let-7f-5p	hsa-miR-132-3p	hsa-miR-29a-3p		
114	SLCO5A1	0.017	0.277	0.114	2	hsa-let-7e-5p/hsa-let-7f-5p	hsa-miR-29a-3p			
115	SOX6	0.015	0.277	0.313	4	hsa-let-7e-5p/hsa-let-7f-5p	hsa-miR-132-3p	hsa-miR-29a-3p	hsa-miR-378f	
116	TMEM2	0.017	0.277	0.114	2	hsa-let-7e-5p/hsa-let-7f-5p	hsa-miR-132-3p			
117	TMEM234	0.004	0.277	0.057	2	hsa-let-7e-5p/hsa-let-7f-5p	hsa-miR-29a-3p			
118	UBTF	0.008	0.277	0.080	2	hsa-miR-1306-5p	hsa-miR-29a-3p			
119	USP38	0.006	0.277	0.152	3	hsa-let-7e-5p/hsa-let-7f-5p	hsa-miR-1306-5p	hsa-miR-132-3p		
120	WARS2	0.006	0.277	0.068	2	hsa-let-7e-5p/hsa-let-7f-5p	hsa-miR-1306-5p			
121	WSCD2	0.011	0.277	0.091	2	hsa-miR-1306-5p	hsa-miR-29a-3p			
122	XPO5	0.021	0.277	0.125	2	hsa-miR-29a-3p	hsa-miR-378f			
123	ZNF282	0.006	0.277	0.068	2	hsa-let-7e-5p/hsa-let-7f-5p	hsa-miR-29a-3p			
124	ZNF652	0.022	0.277	0.347	4	hsa-let-7e-5p/hsa-let-7f-5p	hsa-miR-1306-5p	hsa-miR-132-3p	hsa-miR-378f	

125	CBX5	0.023	0.280	0.352	4	hsa-let-7e-5p/hsa-let-7f-5p	hsa-miR-1306-5p	hsa-miR-29a-3p	hsa-miR-378f	
126	ALS2	0.025	0.283	0.136	2	hsa-miR-29a-3p	hsa-miR-378f			
127	AP1S1	0.025	0.283	0.136	2	hsa-let-7e-5p/hsa-let-7f-5p	hsa-miR-29a-3p			
128	B3GNT1	0.025	0.283	0.136	2	hsa-let-7e-5p/hsa-let-7f-5p	hsa-miR-1306-5p			
129	CBX3	0.025	0.283	0.136	2	hsa-miR-29a-3p	hsa-miR-378f			
130	CDC42SE1	0.025	0.283	0.136	2	hsa-let-7e-5p/hsa-let-7f-5p	hsa-miR-29a-3p			
131	COL1A1	0.025	0.283	0.136	2	hsa-let-7e-5p/hsa-let-7f-5p	hsa-miR-29a-3p			
132	CPM	0.025	0.283	0.136	2	hsa-let-7e-5p/hsa-let-7f-5p	hsa-miR-29a-3p			
133	DCAF8	0.025	0.283	0.136	2	hsa-let-7e-5p/hsa-let-7f-5p	hsa-miR-132-3p			
134	FAM83F	0.025	0.283	0.250	3	hsa-miR-1306-5p	hsa-miR-29a-3p	hsa-miR-378f		
135	FRAS1	0.025	0.283	0.136	2	hsa-let-7e-5p/hsa-let-7f-5p	hsa-miR-29a-3p			
136	GPR37L1	0.025	0.283	0.136	2	hsa-miR-1306-5p	hsa-miR-132-3p			
137	MAPKBP1	0.025	0.283	0.136	2	hsa-miR-132-3p	hsa-miR-29a-3p			
138	NCOR1	0.025	0.283	0.136	2	hsa-let-7e-5p/hsa-let-7f-5p	hsa-miR-132-3p			
139	PIK3CA	0.025	0.283	0.250	3	hsa-let-7e-5p/hsa-let-7f-5p	hsa-miR-132-3p	hsa-miR-29a-3p		
140	PXDN	0.025	0.283	0.136	2	hsa-let-7e-5p/hsa-let-7f-5p	hsa-miR-29a-3p			
141	RNF144B	0.025	0.283	0.136	2	hsa-miR-29a-3p	hsa-miR-378f			
142	SDK1	0.025	0.283	0.136	2	hsa-let-7e-5p/hsa-let-7f-5p	hsa-miR-29a-3p			
143	ZNF362	0.025	0.283	0.136	2	hsa-let-7e-5p/hsa-let-7f-5p	hsa-miR-29a-3p			
144	ABHD5	0.029	0.299	0.148	2	hsa-miR-132-3p	hsa-miR-29a-3p			
145	ADAMTS5	0.040	0.299	0.295	3	hsa-let-7e-5p/hsa-let-7f-5p	hsa-miR-132-3p	hsa-miR-29a-3p		

146	AKAP5	0.034	0.299	0.159	2	hsa-let-7e-5p/hsa-let-7f-5p	hsa-miR-29a-3p			
147	AMMECR1L	0.039	0.299	0.170	2	hsa-let-7e-5p/hsa-let-7f-5p	hsa-miR-29a-3p			
148	ARID1B	0.039	0.299	0.170	2	hsa-miR-132-3p	hsa-miR-29a-3p			
149	B3GALT1	0.044	0.299	0.182	2	hsa-let-7e-5p/hsa-let-7f-5p	hsa-miR-29a-3p			
150	BCL2L2	0.027	0.299	0.258	3	hsa-miR-1306-5p	hsa-miR-29a-3p	hsa-miR-378f		
151	CLMN	0.039	0.299	0.170	2	hsa-miR-132-3p	hsa-miR-29a-3p			
152	CNOT2	0.030	0.299	0.265	3	hsa-let-7e-5p/hsa-let-7f-5p	hsa-miR-1306-5p	hsa-miR-132-3p		
153	COLEC10	0.034	0.299	0.159	2	hsa-miR-1306-5p	hsa-miR-132-3p			
154	DCX	0.035	0.299	0.280	3	hsa-let-7e-5p/hsa-let-7f-5p	hsa-miR-29a-3p	hsa-miR-378f		
155	DNAH10OS	0.034	0.299	0.159	2	hsa-let-7e-5p/hsa-let-7f-5p	hsa-miR-1306-5p			
156	DTX4	0.044	0.299	0.182	2	hsa-let-7e-5p/hsa-let-7f-5p	hsa-miR-29a-3p			
157	E2F5	0.029	0.299	0.148	2	hsa-let-7e-5p/hsa-let-7f-5p	hsa-miR-132-3p			
158	FAM167A	0.034	0.299	0.159	2	hsa-miR-132-3p	hsa-miR-29a-3p			
159	FAM84B	0.034	0.299	0.159	2	hsa-let-7e-5p/hsa-let-7f-5p	hsa-miR-29a-3p			
160	FBXL19	0.039	0.299	0.170	2	hsa-let-7e-5p/hsa-let-7f-5p	hsa-miR-1306-5p			
161	GAS7	0.044	0.299	0.182	2	hsa-let-7e-5p/hsa-let-7f-5p	hsa-miR-29a-3p			
162	GOPC	0.034	0.299	0.159	2	hsa-let-7e-5p/hsa-let-7f-5p	hsa-miR-29a-3p			
163	HEY2	0.039	0.299	0.170	2	hsa-miR-1306-5p	hsa-miR-29a-3p			
164	HIP1	0.044	0.299	0.182	2	hsa-let-7e-5p/hsa-let-7f-5p	hsa-miR-29a-3p			
165	HOXD12	0.029	0.299	0.148	2	hsa-miR-132-3p	hsa-miR-29a-3p			
166	IGF1	0.032	0.299	0.273	3	hsa-let-7e-5p/hsa-let-7f-5p	hsa-miR-29a-3p	hsa-miR-378f		

167	ISL1	0.029	0.299	0.148	2	hsa-miR-132-3p	hsa-miR-29a-3p			
168	KCNA6	0.029	0.299	0.148	2	hsa-let-7e-5p/hsa-let-7f-5p	hsa-miR-132-3p			
169	KCTD15	0.039	0.299	0.170	2	hsa-let-7e-5p/hsa-let-7f-5p	hsa-miR-29a-3p			
170	KIAA1644	0.034	0.299	0.159	2	hsa-miR-1306-5p	hsa-miR-29a-3p			
171	LBH	0.034	0.299	0.159	2	hsa-let-7e-5p/hsa-let-7f-5p	hsa-miR-1306-5p			
172	LMX1A	0.039	0.299	0.170	2	hsa-let-7e-5p/hsa-let-7f-5p	hsa-miR-29a-3p			
173	MAP4K4	0.044	0.299	0.182	2	hsa-let-7e-5p/hsa-let-7f-5p	hsa-miR-29a-3p			
174	MIEF1	0.039	0.299	0.170	2	hsa-let-7e-5p/hsa-let-7f-5p	hsa-miR-29a-3p			
175	MTMR9	0.044	0.299	0.182	2	hsa-miR-1306-5p	hsa-miR-29a-3p			
176	NANOS1	0.039	0.299	0.170	2	hsa-miR-1306-5p	hsa-miR-29a-3p			
177	NHLRC2	0.044	0.299	0.182	2	hsa-let-7e-5p/hsa-let-7f-5p	hsa-miR-29a-3p			
178	PCYT1B	0.044	0.299	0.182	2	hsa-let-7e-5p/hsa-let-7f-5p	hsa-miR-29a-3p			
179	PDP2	0.044	0.299	0.182	2	hsa-let-7e-5p/hsa-let-7f-5p	hsa-miR-29a-3p			
180	PPP1R15B	0.044	0.299	0.182	2	hsa-let-7e-5p/hsa-let-7f-5p	hsa-miR-29a-3p			
181	PRR14L	0.027	0.299	0.258	3	hsa-let-7e-5p/hsa-let-7f-5p	hsa-miR-132-3p	hsa-miR-29a-3p		
182	PTPRD	0.043	0.299	0.303	3	hsa-let-7e-5p/hsa-let-7f-5p	hsa-miR-132-3p	hsa-miR-29a-3p		
183	PTRH2	0.029	0.299	0.148	2	hsa-miR-29a-3p	hsa-miR-378f			
184	PXMP4	0.044	0.299	0.182	2	hsa-let-7e-5p/hsa-let-7f-5p	hsa-miR-29a-3p			
185	RBFOX1	0.029	0.299	0.148	2	hsa-let-7e-5p/hsa-let-7f-5p	hsa-miR-132-3p			
186	RBMS2	0.039	0.299	0.170	2	hsa-let-7e-5p/hsa-let-7f-5p	hsa-miR-29a-3p			
187	REL	0.034	0.299	0.159	2	hsa-let-7e-5p/hsa-let-7f-5p	hsa-miR-29a-3p			

188	SCN5A	0.034	0.299	0.159	2	hsa-let-7e-5p/hsa-let-7f-5p	hsa-miR-378f			
189	SCYL3	0.044	0.299	0.182	2	hsa-let-7e-5p/hsa-let-7f-5p	hsa-miR-132-3p			
190	SETD5	0.039	0.299	0.170	2	hsa-miR-132-3p	hsa-miR-29a-3p			
191	SLC30A6	0.039	0.299	0.170	2	hsa-let-7e-5p/hsa-let-7f-5p	hsa-miR-132-3p			
192	SLC39A13	0.039	0.299	0.170	2	hsa-miR-1306-5p	hsa-miR-29a-3p			
193	SPAST	0.029	0.299	0.148	2	hsa-miR-132-3p	hsa-miR-29a-3p			
194	STAT3	0.034	0.299	0.159	2	hsa-let-7e-5p/hsa-let-7f-5p	hsa-miR-29a-3p			
195	STX17	0.039	0.299	0.170	2	hsa-let-7e-5p/hsa-let-7f-5p	hsa-miR-29a-3p			
196	SUB1	0.039	0.299	0.170	2	hsa-let-7e-5p/hsa-let-7f-5p	hsa-miR-29a-3p			
197	SYT7	0.044	0.299	0.182	2	hsa-let-7e-5p/hsa-let-7f-5p	hsa-miR-29a-3p			
198	TADA2B	0.044	0.299	0.182	2	hsa-miR-132-3p	hsa-miR-29a-3p			
199	TMED5	0.039	0.299	0.170	2	hsa-let-7e-5p/hsa-let-7f-5p	hsa-miR-132-3p			
200	TMEM255A	0.039	0.299	0.170	2	hsa-let-7e-5p/hsa-let-7f-5p	hsa-miR-29a-3p			
201	TMTC3	0.039	0.299	0.170	2	hsa-let-7e-5p/hsa-let-7f-5p	hsa-miR-29a-3p			
202	TNFAIP3	0.034	0.299	0.159	2	hsa-let-7e-5p/hsa-let-7f-5p	hsa-miR-29a-3p			
203	TSPAN9	0.039	0.299	0.170	2	hsa-miR-1306-5p	hsa-miR-29a-3p			
204	WDR37	0.030	0.299	0.265	3	hsa-let-7e-5p/hsa-let-7f-5p	hsa-miR-29a-3p	hsa-miR-378f		
205	WNT9B	0.044	0.299	0.182	2	hsa-let-7e-5p/hsa-let-7f-5p	hsa-miR-1306-5p			
206	XKRX	0.029	0.299	0.148	2	hsa-let-7e-5p/hsa-let-7f-5p	hsa-miR-29a-3p			
207	YY1	0.032	0.299	0.273	3	hsa-let-7e-5p/hsa-let-7f-5p	hsa-miR-29a-3p	hsa-miR-378f		
208	ZNF512B	0.034	0.299	0.159	2	hsa-let-7e-5p/hsa-let-7f-5p	hsa-miR-29a-3p			

209	ZNF641	0.039	0.299	0.170	2	hsa-let-7e-5p/hsa-let-7f-5p	hsa-miR-29a-3p			
210	KCNJ6	0.046	0.304	0.311	3	hsa-let-7e-5p/hsa-let-7f-5p	hsa-miR-132-3p	hsa-miR-29a-3p		
211	KPNA1	0.046	0.304	0.311	3	hsa-let-7e-5p/hsa-let-7f-5p	hsa-miR-132-3p	hsa-miR-29a-3p		
212	CCDC117	0.049	0.316	0.193	2	hsa-miR-132-3p	hsa-miR-29a-3p			
213	DCUN1D3	0.049	0.316	0.318	3	hsa-let-7e-5p/hsa-let-7f-5p	hsa-miR-1306-5p	hsa-miR-132-3p		
214	FBXO21	0.049	0.316	0.193	2	hsa-let-7e-5p/hsa-let-7f-5p	hsa-miR-132-3p			
215	GNS	0.049	0.316	0.193	2	hsa-let-7e-5p/hsa-let-7f-5p	hsa-miR-29a-3p			
216	IPO9	0.049	0.316	0.193	2	hsa-let-7e-5p/hsa-let-7f-5p	hsa-miR-378f			
217	KIAA1958	0.049	0.316	0.193	2	hsa-let-7e-5p/hsa-let-7f-5p	hsa-miR-132-3p			
218	MEIS1	0.049	0.316	0.318	3	hsa-let-7e-5p/hsa-let-7f-5p	hsa-miR-1306-5p	hsa-miR-132-3p		
219	MYCN	0.049	0.316	0.193	2	hsa-let-7e-5p/hsa-let-7f-5p	hsa-miR-29a-3p			
220	NREP	0.049	0.316	0.193	2	hsa-miR-132-3p	hsa-miR-29a-3p			
221	SEMA4G	0.049	0.316	0.193	2	hsa-let-7e-5p/hsa-let-7f-5p	hsa-miR-132-3p			
222	SLK	0.049	0.316	0.193	2	hsa-let-7e-5p/hsa-let-7f-5p	hsa-miR-29a-3p			
223	XKR4	0.049	0.316	0.318	3	hsa-let-7e-5p/hsa-let-7f-5p	hsa-miR-1306-5p	hsa-miR-29a-3p		
224	ZBTB5	0.049	0.316	0.193	2	hsa-let-7e-5p/hsa-let-7f-5p	hsa-miR-29a-3p			
225	BMP2	0.067	0.321	0.227	2	hsa-let-7e-5p/hsa-let-7f-5p	hsa-miR-378f			
226	C11orf57	0.055	0.321	0.205	2	hsa-let-7e-5p/hsa-let-7f-5p	hsa-miR-29a-3p			
227	C16orf87	0.067	0.321	0.227	2	hsa-miR-132-3p	hsa-miR-29a-3p			
228	COL4A4	0.055	0.321	0.205	2	hsa-miR-132-3p	hsa-miR-29a-3p			
229	CSRNP3	0.059	0.321	0.341	3	hsa-let-7e-5p/hsa-let-7f-5p	hsa-miR-132-3p	hsa-miR-378f		

230	CYTH3	0.055	0.321	0.205	2	hsa-let-7e-5p/hsa-let-7f-5p	hsa-miR-29a-3p			
231	DCUN1D4	0.067	0.321	0.227	2	hsa-miR-132-3p	hsa-miR-29a-3p			
232	DDI2	0.063	0.321	0.348	3	hsa-let-7e-5p/hsa-let-7f-5p	hsa-miR-29a-3p	hsa-miR-378f		
233	DENND6A	0.067	0.321	0.227	2	hsa-let-7e-5p/hsa-let-7f-5p	hsa-miR-29a-3p			
234	DNAJA2	0.055	0.321	0.205	2	hsa-let-7e-5p/hsa-let-7f-5p	hsa-miR-132-3p			
235	DNAJB14	0.055	0.321	0.205	2	hsa-let-7e-5p/hsa-let-7f-5p	hsa-miR-29a-3p			
236	DNMT3A	0.063	0.321	0.348	3	hsa-miR-1306-5p	hsa-miR-132-3p	hsa-miR-29a-3p		
237	ENTPD7	0.067	0.321	0.227	2	hsa-let-7e-5p/hsa-let-7f-5p	hsa-miR-29a-3p			
238	FOXP1	0.056	0.321	0.333	3	hsa-let-7e-5p/hsa-let-7f-5p	hsa-miR-132-3p	hsa-miR-29a-3p		
239	JMY	0.055	0.321	0.205	2	hsa-miR-132-3p	hsa-miR-29a-3p			
240	KIF21B	0.055	0.321	0.205	2	hsa-let-7e-5p/hsa-let-7f-5p	hsa-miR-132-3p			
241	KIF26B	0.067	0.321	0.227	2	hsa-miR-29a-3p	hsa-miR-378f			
242	LPPR5	0.055	0.321	0.205	2	hsa-miR-1306-5p	hsa-miR-29a-3p			
243	MOB1B	0.061	0.321	0.216	2	hsa-miR-1306-5p	hsa-miR-29a-3p			
244	OSBPL3	0.055	0.321	0.205	2	hsa-let-7e-5p/hsa-let-7f-5p	hsa-miR-29a-3p			
245	PITPNM3	0.061	0.321	0.216	2	hsa-let-7e-5p/hsa-let-7f-5p	hsa-miR-29a-3p			
246	PLEKHA8	0.067	0.321	0.227	2	hsa-let-7e-5p/hsa-let-7f-5p	hsa-miR-29a-3p			
247	PPP3CA	0.067	0.321	0.227	2	hsa-let-7e-5p/hsa-let-7f-5p	hsa-miR-132-3p			
248	PRKAB2	0.061	0.321	0.216	2	hsa-let-7e-5p/hsa-let-7f-5p	hsa-miR-29a-3p			
249	RAB6B	0.055	0.321	0.205	2	hsa-miR-132-3p	hsa-miR-29a-3p			
250	RCC2	0.055	0.321	0.205	2	hsa-miR-29a-3p	hsa-miR-378f			

251	SLC39A9	0.055	0.321	0.205	2	hsa-miR-1306-5p	hsa-miR-29a-3p			
252	SMARCC1	0.055	0.321	0.205	2	hsa-let-7e-5p/hsa-let-7f-5p	hsa-miR-29a-3p			
253	SPRY1	0.067	0.321	0.227	2	hsa-miR-132-3p	hsa-miR-29a-3p			
254	TMEM178B	0.059	0.321	0.341	3	hsa-let-7e-5p/hsa-let-7f-5p	hsa-miR-132-3p	hsa-miR-29a-3p		
255	TOB2	0.055	0.321	0.205	2	hsa-let-7e-5p/hsa-let-7f-5p	hsa-miR-378f			
256	VAT1	0.067	0.321	0.227	2	hsa-miR-1306-5p	hsa-miR-378f			
257	ZBTB8B	0.055	0.321	0.205	2	hsa-let-7e-5p/hsa-let-7f-5p	hsa-miR-29a-3p			
258	ZCCHC11	0.055	0.321	0.205	2	hsa-let-7e-5p/hsa-let-7f-5p	hsa-miR-132-3p			
259	ZNF644	0.067	0.321	0.227	2	hsa-let-7e-5p/hsa-let-7f-5p	hsa-miR-132-3p			
260	ZNF710	0.055	0.321	0.205	2	hsa-let-7e-5p/hsa-let-7f-5p	hsa-miR-1306-5p			
261	AC068987.1	0.079	0.325	0.250	2	hsa-let-7e-5p/hsa-let-7f-5p	hsa-miR-1306-5p			
262	ARL5B	0.073	0.325	0.239	2	hsa-let-7e-5p/hsa-let-7f-5p	hsa-miR-29a-3p			
263	ATP2B4	0.073	0.325	0.239	2	hsa-let-7e-5p/hsa-let-7f-5p	hsa-miR-29a-3p			
264	CAMKK2	0.073	0.325	0.239	2	hsa-miR-29a-3p	hsa-miR-378f			
265	CCDC71L	0.079	0.325	0.250	2	hsa-let-7e-5p/hsa-let-7f-5p	hsa-miR-132-3p			
266	CFL2	0.075	0.325	0.371	3	hsa-let-7e-5p/hsa-let-7f-5p	hsa-miR-132-3p	hsa-miR-29a-3p		
267	CNIH1	0.073	0.325	0.239	2	hsa-miR-132-3p	hsa-miR-29a-3p			
268	CREB5	0.073	0.325	0.239	2	hsa-miR-132-3p	hsa-miR-29a-3p			
269	DAAM1	0.079	0.325	0.250	2	hsa-miR-132-3p	hsa-miR-29a-3p			
270	FBXO42	0.073	0.325	0.239	2	hsa-miR-132-3p	hsa-miR-29a-3p			
271	FOXO3	0.073	0.325	0.239	2	hsa-miR-132-3p	hsa-miR-29a-3p			

272	GABPB2	0.086	0.325	0.261	2	hsa-miR-132-3p	hsa-miR-29a-3p			
273	GHR	0.073	0.325	0.239	2	hsa-let-7e-5p/hsa-let-7f-5p	hsa-miR-132-3p			
274	HMGA2	0.073	0.325	0.239	2	hsa-let-7e-5p/hsa-let-7f-5p	hsa-miR-132-3p			
275	HMGCS1	0.073	0.325	0.239	2	hsa-miR-1306-5p	hsa-miR-29a-3p			
276	JOSD1	0.079	0.325	0.250	2	hsa-let-7e-5p/hsa-let-7f-5p	hsa-miR-29a-3p			
277	KCNC2	0.073	0.325	0.239	2	hsa-let-7e-5p/hsa-let-7f-5p	hsa-miR-29a-3p			
278	KDM5B	0.073	0.325	0.239	2	hsa-miR-132-3p	hsa-miR-29a-3p			
279	MAPK8	0.079	0.325	0.250	2	hsa-let-7e-5p/hsa-let-7f-5p	hsa-miR-29a-3p			
280	MBD2	0.086	0.325	0.261	2	hsa-let-7e-5p/hsa-let-7f-5p	hsa-miR-1306-5p			
281	MBTD1	0.086	0.325	0.261	2	hsa-let-7e-5p/hsa-let-7f-5p	hsa-miR-29a-3p			
282	MIB1	0.073	0.325	0.239	2	hsa-let-7e-5p/hsa-let-7f-5p	hsa-miR-29a-3p			
283	MLXIP	0.073	0.325	0.239	2	hsa-let-7e-5p/hsa-let-7f-5p	hsa-miR-29a-3p			
284	NKAP	0.086	0.325	0.261	2	hsa-let-7e-5p/hsa-let-7f-5p	hsa-miR-1306-5p			
285	NRAS	0.079	0.325	0.250	2	hsa-let-7e-5p/hsa-let-7f-5p	hsa-miR-29a-3p			
286	ONECUT1	0.073	0.325	0.239	2	hsa-let-7e-5p/hsa-let-7f-5p	hsa-miR-29a-3p			
287	OTUD3	0.086	0.325	0.261	2	hsa-let-7e-5p/hsa-let-7f-5p	hsa-miR-132-3p			
288	PHACTR2	0.086	0.325	0.261	2	hsa-let-7e-5p/hsa-let-7f-5p	hsa-miR-29a-3p			
289	PITPNA	0.086	0.325	0.261	2	hsa-miR-29a-3p	hsa-miR-378f			
290	RDX	0.079	0.325	0.250	2	hsa-let-7e-5p/hsa-let-7f-5p	hsa-miR-132-3p			
291	REST	0.073	0.325	0.239	2	hsa-miR-29a-3p	hsa-miR-378f			
292	SLC16A10	0.086	0.325	0.261	2	hsa-let-7e-5p/hsa-let-7f-5p	hsa-miR-29a-3p			

293	SLC6A1	0.073	0.325	0.239	2	hsa-let-7e-5p/hsa-let-7f-5p	hsa-miR-132-3p			
294	SMC1A	0.079	0.325	0.250	2	hsa-let-7e-5p/hsa-let-7f-5p	hsa-miR-1306-5p			
295	SNIP1	0.073	0.325	0.239	2	hsa-miR-132-3p	hsa-miR-29a-3p			
296	STX16	0.079	0.325	0.250	2	hsa-miR-132-3p	hsa-miR-29a-3p			
297	TFCP2L1	0.073	0.325	0.239	2	hsa-miR-1306-5p	hsa-miR-378f			
298	UBFD1	0.073	0.325	0.239	2	hsa-miR-1306-5p	hsa-miR-29a-3p			
299	VANGL1	0.086	0.325	0.261	2	hsa-miR-29a-3p	hsa-miR-378f			
300	VASH2	0.086	0.325	0.261	2	hsa-let-7e-5p/hsa-let-7f-5p	hsa-miR-29a-3p			
301	XKR7	0.079	0.325	0.250	2	hsa-let-7e-5p/hsa-let-7f-5p	hsa-miR-29a-3p			
302	XRN1	0.079	0.325	0.250	2	hsa-let-7e-5p/hsa-let-7f-5p	hsa-miR-29a-3p			
303	ZFYVE26	0.073	0.325	0.239	2	hsa-let-7e-5p/hsa-let-7f-5p	hsa-miR-29a-3p			
304	ATAD2B	0.108	0.336	0.295	2	hsa-let-7e-5p/hsa-let-7f-5p	hsa-miR-29a-3p			
305	BAHD1	0.100	0.336	0.284	2	hsa-let-7e-5p/hsa-let-7f-5p	hsa-miR-29a-3p			
306	BCL9L	0.093	0.336	0.273	2	hsa-miR-1306-5p	hsa-miR-29a-3p			
307	C11orf87	0.093	0.336	0.273	2	hsa-miR-132-3p	hsa-miR-29a-3p			
308	CAMSAP2	0.100	0.336	0.284	2	hsa-miR-132-3p	hsa-miR-29a-3p			
309	CBX6	0.093	0.336	0.273	2	hsa-miR-1306-5p	hsa-miR-29a-3p			
310	DCAF12	0.108	0.336	0.295	2	hsa-miR-29a-3p	hsa-miR-378f			
311	DIAPH2	0.100	0.336	0.284	2	hsa-let-7e-5p/hsa-let-7f-5p	hsa-miR-29a-3p			
312	DICER1	0.100	0.336	0.284	2	hsa-let-7e-5p/hsa-let-7f-5p	hsa-miR-29a-3p			
313	FBXW7	0.108	0.336	0.295	2	hsa-miR-132-3p	hsa-miR-29a-3p			

314	FMNL3	0.093	0.336	0.273	2	hsa-miR-1306-5p	hsa-miR-29a-3p			
315	FOXN3	0.100	0.336	0.284	2	hsa-miR-132-3p	hsa-miR-29a-3p			
316	IGF1R	0.108	0.336	0.295	2	hsa-let-7e-5p/hsa-let-7f-5p	hsa-miR-378f			
317	LIN28B	0.100	0.336	0.284	2	hsa-let-7e-5p/hsa-let-7f-5p	hsa-miR-132-3p			
318	MAP3K9	0.100	0.336	0.284	2	hsa-let-7e-5p/hsa-let-7f-5p	hsa-miR-132-3p			
319	PDIK1L	0.093	0.336	0.273	2	hsa-miR-29a-3p	hsa-miR-378f			
320	PGM2L1	0.100	0.336	0.284	2	hsa-let-7e-5p/hsa-let-7f-5p	hsa-miR-1306-5p			
321	RGS7BP	0.108	0.336	0.295	2	hsa-miR-1306-5p	hsa-miR-132-3p			
322	RNF165	0.100	0.336	0.284	2	hsa-let-7e-5p/hsa-let-7f-5p	hsa-miR-29a-3p			
323	RP11-766F14.2	0.108	0.336	0.295	2	hsa-let-7e-5p/hsa-let-7f-5p	hsa-miR-29a-3p			
324	SIRT1	0.093	0.336	0.273	2	hsa-miR-132-3p	hsa-miR-29a-3p			
325	SP8	0.100	0.336	0.284	2	hsa-let-7e-5p/hsa-let-7f-5p	hsa-miR-1306-5p			
326	SRGAP2	0.100	0.336	0.284	2	hsa-miR-132-3p	hsa-miR-29a-3p			
327	SRSF10	0.096	0.336	0.409	3	hsa-miR-132-3p	hsa-miR-29a-3p	hsa-miR-378f		
328	UCK2	0.093	0.336	0.273	2	hsa-miR-29a-3p	hsa-miR-378f			
329	WASF2	0.108	0.336	0.295	2	hsa-miR-1306-5p	hsa-miR-29a-3p			
330	YPEL2	0.100	0.336	0.284	2	hsa-let-7e-5p/hsa-let-7f-5p	hsa-miR-29a-3p			
331	ZBTB37	0.108	0.336	0.295	2	hsa-let-7e-5p/hsa-let-7f-5p	hsa-miR-29a-3p			
332	PDE7A	0.111	0.339	0.432	3	hsa-miR-1306-5p	hsa-miR-132-3p	hsa-miR-29a-3p		
333	C1orf21	0.115	0.347	0.307	2	hsa-miR-29a-3p	hsa-miR-378f			
334	CALN1	0.123	0.347	0.318	2	hsa-let-7e-5p/hsa-let-7f-5p	hsa-miR-378f			

335	CAMK1D	0.123	0.347	0.318	2	hsa-miR-29a-3p	hsa-miR-378f			
336	CCDC88A	0.130	0.347	0.330	2	hsa-miR-132-3p	hsa-miR-29a-3p			
337	CEP85L	0.123	0.347	0.318	2	hsa-let-7e-5p/hsa-let-7f-5p	hsa-miR-29a-3p			
338	CYB561D1	0.123	0.347	0.318	2	hsa-let-7e-5p/hsa-let-7f-5p	hsa-miR-29a-3p			
339	DNAL1	0.123	0.347	0.318	2	hsa-let-7e-5p/hsa-let-7f-5p	hsa-miR-29a-3p			
340	FRMPD4	0.115	0.347	0.307	2	hsa-miR-132-3p	hsa-miR-378f			
341	GPCPD1	0.130	0.347	0.330	2	hsa-let-7e-5p/hsa-let-7f-5p	hsa-miR-29a-3p			
342	GRAMD1B	0.123	0.347	0.318	2	hsa-let-7e-5p/hsa-let-7f-5p	hsa-miR-29a-3p			
343	GRSF1	0.115	0.347	0.307	2	hsa-miR-132-3p	hsa-miR-378f			
344	KCNC3	0.123	0.347	0.318	2	hsa-let-7e-5p/hsa-let-7f-5p	hsa-miR-29a-3p			
345	LPGAT1	0.115	0.347	0.307	2	hsa-let-7e-5p/hsa-let-7f-5p	hsa-miR-29a-3p			
346	MAFG	0.115	0.347	0.307	2	hsa-miR-1306-5p	hsa-miR-29a-3p			
347	MAP3K3	0.130	0.347	0.330	2	hsa-let-7e-5p/hsa-let-7f-5p	hsa-miR-132-3p			
348	MAPK6	0.130	0.347	0.330	2	hsa-let-7e-5p/hsa-let-7f-5p	hsa-miR-1306-5p			
349	MGA	0.123	0.347	0.318	2	hsa-let-7e-5p/hsa-let-7f-5p	hsa-miR-29a-3p			
350	PRPF40A	0.115	0.347	0.307	2	hsa-miR-1306-5p	hsa-miR-29a-3p			
351	RALGPS1	0.123	0.347	0.318	2	hsa-let-7e-5p/hsa-let-7f-5p	hsa-miR-29a-3p			
352	ROBO1	0.115	0.347	0.307	2	hsa-let-7e-5p/hsa-let-7f-5p	hsa-miR-29a-3p			
353	SYT2	0.130	0.347	0.330	2	hsa-let-7e-5p/hsa-let-7f-5p	hsa-miR-29a-3p			
354	TET3	0.121	0.347	0.447	3	hsa-let-7e-5p/hsa-let-7f-5p	hsa-miR-1306-5p	hsa-miR-29a-3p		
355	THBS1	0.123	0.347	0.318	2	hsa-let-7e-5p/hsa-let-7f-5p	hsa-miR-132-3p			

356	TMEM65	0.115	0.347	0.307	2	hsa-let-7e-5p/hsa-let-7f-5p	hsa-miR-29a-3p			
357	ACVR2A	0.146	0.354	0.352	2	hsa-let-7e-5p/hsa-let-7f-5p	hsa-miR-29a-3p			
358	ARMC8	0.146	0.354	0.352	2	hsa-let-7e-5p/hsa-let-7f-5p	hsa-miR-29a-3p			
359	ARPP19	0.146	0.354	0.352	2	hsa-let-7e-5p/hsa-let-7f-5p	hsa-miR-29a-3p			
360	BTBD7	0.146	0.354	0.352	2	hsa-miR-132-3p	hsa-miR-29a-3p			
361	C16orf72	0.146	0.354	0.352	2	hsa-miR-29a-3p	hsa-miR-378f			
362	CNOT6L	0.138	0.354	0.341	2	hsa-let-7e-5p/hsa-let-7f-5p	hsa-miR-1306-5p			
363	CTDSPL2	0.143	0.354	0.477	3	hsa-let-7e-5p/hsa-let-7f-5p	hsa-miR-132-3p	hsa-miR-29a-3p		
364	DYNC1LI2	0.146	0.354	0.352	2	hsa-miR-132-3p	hsa-miR-378f			
365	GABBR2	0.138	0.354	0.341	2	hsa-let-7e-5p/hsa-let-7f-5p	hsa-miR-1306-5p			
366	HAPLN1	0.138	0.354	0.341	2	hsa-miR-132-3p	hsa-miR-29a-3p			
367	HCFC2	0.138	0.354	0.341	2	hsa-let-7e-5p/hsa-let-7f-5p	hsa-miR-1306-5p			
368	MAP2K6	0.146	0.354	0.352	2	hsa-miR-29a-3p	hsa-miR-378f			
369	RAB30	0.138	0.354	0.341	2	hsa-let-7e-5p/hsa-let-7f-5p	hsa-miR-29a-3p			
370	RNF152	0.146	0.354	0.352	2	hsa-let-7e-5p/hsa-let-7f-5p	hsa-miR-29a-3p			
371	RNF217	0.138	0.354	0.341	2	hsa-let-7e-5p/hsa-let-7f-5p	hsa-miR-29a-3p			
372	RUNX1T1	0.137	0.354	0.470	3	hsa-let-7e-5p/hsa-let-7f-5p	hsa-miR-132-3p	hsa-miR-29a-3p		
373	SRGAP1	0.138	0.354	0.341	2	hsa-let-7e-5p/hsa-let-7f-5p	hsa-miR-132-3p			
374	ETNK1	0.155	0.360	0.364	2	hsa-let-7e-5p/hsa-let-7f-5p	hsa-miR-132-3p			
375	FNDC3B	0.155	0.360	0.364	2	hsa-let-7e-5p/hsa-let-7f-5p	hsa-miR-1306-5p			
376	GAB1	0.155	0.360	0.364	2	hsa-miR-132-3p	hsa-miR-29a-3p			

377	GSK3B	0.155	0.360	0.364	2	hsa-miR-132-3p	hsa-miR-29a-3p			
378	HELZ	0.155	0.360	0.364	2	hsa-let-7e-5p/hsa-let-7f-5p	hsa-miR-29a-3p			
379	LNPEP	0.155	0.360	0.364	2	hsa-miR-29a-3p	hsa-miR-378f			
380	PALM2	0.155	0.360	0.364	2	hsa-miR-132-3p	hsa-miR-29a-3p			
381	PAPOLG	0.155	0.360	0.364	2	hsa-miR-29a-3p	hsa-miR-378f			
382	PEG10	0.155	0.360	0.364	2	hsa-let-7e-5p/hsa-let-7f-5p	hsa-miR-29a-3p			
383	PRKAA2	0.155	0.360	0.364	2	hsa-let-7e-5p/hsa-let-7f-5p	hsa-miR-132-3p			
384	SRGAP3	0.155	0.360	0.364	2	hsa-let-7e-5p/hsa-let-7f-5p	hsa-miR-132-3p			
385	ZNF516	0.155	0.360	0.364	2	hsa-let-7e-5p/hsa-let-7f-5p	hsa-miR-132-3p			
386	ZNF609	0.155	0.360	0.364	2	hsa-miR-1306-5p	hsa-miR-29a-3p			
387	BSN	0.163	0.367	0.375	2	hsa-let-7e-5p/hsa-let-7f-5p	hsa-miR-132-3p			
388	FAXC	0.163	0.367	0.375	2	hsa-let-7e-5p/hsa-let-7f-5p	hsa-miR-29a-3p			
389	HLF	0.163	0.367	0.375	2	hsa-let-7e-5p/hsa-let-7f-5p	hsa-miR-29a-3p			
390	HNRNPA1	0.163	0.367	0.375	2	hsa-let-7e-5p/hsa-let-7f-5p	hsa-miR-378f			
391	KDM2A	0.163	0.367	0.375	2	hsa-miR-1306-5p	hsa-miR-29a-3p			
392	MAPK1IP1L	0.163	0.367	0.375	2	hsa-let-7e-5p/hsa-let-7f-5p	hsa-miR-378f			
393	MXD1	0.163	0.367	0.375	2	hsa-let-7e-5p/hsa-let-7f-5p	hsa-miR-29a-3p			
394	NSD1	0.163	0.367	0.375	2	hsa-let-7e-5p/hsa-let-7f-5p	hsa-miR-29a-3p			
395	SOCS7	0.163	0.367	0.375	2	hsa-let-7e-5p/hsa-let-7f-5p	hsa-miR-29a-3p			
396	TACC1	0.163	0.367	0.375	2	hsa-miR-132-3p	hsa-miR-29a-3p			
397	CCNJ	0.172	0.367	0.386	2	hsa-let-7e-5p/hsa-let-7f-5p	hsa-miR-29a-3p			

398	CRK	0.172	0.367	0.386	2	hsa-let-7e-5p/hsa-let-7f-5p	hsa-miR-132-3p			
399	EPC1	0.172	0.367	0.386	2	hsa-miR-132-3p	hsa-miR-29a-3p			
400	FZD5	0.172	0.367	0.386	2	hsa-miR-29a-3p	hsa-miR-378f			
401	PHF15	0.172	0.367	0.386	2	hsa-miR-1306-5p	hsa-miR-378f			
402	PLAGL2	0.172	0.367	0.386	2	hsa-let-7e-5p/hsa-let-7f-5p	hsa-miR-378f			
403	TET2	0.172	0.367	0.386	2	hsa-let-7e-5p/hsa-let-7f-5p	hsa-miR-29a-3p			
404	TRAF3	0.172	0.367	0.386	2	hsa-miR-29a-3p	hsa-miR-378f			
405	AKIRIN1	0.180	0.374	0.398	2	hsa-miR-1306-5p	hsa-miR-132-3p			
406	BBX	0.189	0.374	0.409	2	hsa-let-7e-5p/hsa-let-7f-5p	hsa-miR-132-3p			
407	BRWD1	0.180	0.374	0.398	2	hsa-miR-132-3p	hsa-miR-29a-3p			
408	ELMSAN1	0.180	0.374	0.398	2	hsa-miR-132-3p	hsa-miR-29a-3p			
409	INO80D	0.180	0.374	0.398	2	hsa-let-7e-5p/hsa-let-7f-5p	hsa-miR-29a-3p			
410	NFIC	0.189	0.374	0.409	2	hsa-miR-1306-5p	hsa-miR-29a-3p			
411	NLK	0.189	0.374	0.409	2	hsa-let-7e-5p/hsa-let-7f-5p	hsa-miR-132-3p			
412	PPARGC1A	0.189	0.374	0.409	2	hsa-let-7e-5p/hsa-let-7f-5p	hsa-miR-29a-3p			
413	THRA	0.189	0.374	0.409	2	hsa-let-7e-5p/hsa-let-7f-5p	hsa-miR-29a-3p			
414	APC	0.198	0.381	0.420	2	hsa-miR-1306-5p	hsa-miR-29a-3p			
415	CLCN5	0.207	0.381	0.432	2	hsa-let-7e-5p/hsa-let-7f-5p	hsa-miR-29a-3p			
416	DYNLL2	0.198	0.381	0.420	2	hsa-miR-1306-5p	hsa-miR-132-3p			
417	GABRB1	0.207	0.381	0.432	2	hsa-let-7e-5p/hsa-let-7f-5p	hsa-miR-29a-3p			
418	H3F3B	0.198	0.381	0.420	2	hsa-miR-132-3p	hsa-miR-378f			

419	KCNK10	0.198	0.381	0.420	2	hsa-miR-1306-5p	hsa-miR-29a-3p			
420	MED12L	0.198	0.381	0.420	2	hsa-miR-29a-3p	hsa-miR-378f			
421	MEF2D	0.198	0.381	0.420	2	hsa-let-7e-5p/hsa-let-7f-5p	hsa-miR-378f			
422	MEIS2	0.198	0.381	0.420	2	hsa-let-7e-5p/hsa-let-7f-5p	hsa-miR-132-3p			
423	MEX3A	0.198	0.381	0.420	2	hsa-let-7e-5p/hsa-let-7f-5p	hsa-miR-132-3p			
424	RBFOX2	0.207	0.381	0.432	2	hsa-let-7e-5p/hsa-let-7f-5p	hsa-miR-29a-3p			
425	SMAD2	0.198	0.381	0.420	2	hsa-let-7e-5p/hsa-let-7f-5p	hsa-miR-132-3p			
426	ZNF24	0.198	0.381	0.420	2	hsa-let-7e-5p/hsa-let-7f-5p	hsa-miR-29a-3p			
427	AKT3	0.216	0.390	0.443	2	hsa-miR-132-3p	hsa-miR-29a-3p			
428	BMPR1A	0.216	0.390	0.443	2	hsa-let-7e-5p/hsa-let-7f-5p	hsa-miR-132-3p			
429	C20orf112	0.216	0.390	0.443	2	hsa-let-7e-5p/hsa-let-7f-5p	hsa-miR-29a-3p			
430	GMFB	0.216	0.390	0.443	2	hsa-miR-132-3p	hsa-miR-29a-3p			
431	KIAA2022	0.216	0.390	0.443	2	hsa-let-7e-5p/hsa-let-7f-5p	hsa-miR-29a-3p			
432	LARP4B	0.216	0.390	0.443	2	hsa-miR-1306-5p	hsa-miR-29a-3p			
433	RBMS1	0.216	0.390	0.443	2	hsa-let-7e-5p/hsa-let-7f-5p	hsa-miR-378f			
434	RSF1	0.216	0.390	0.443	2	hsa-let-7e-5p/hsa-let-7f-5p	hsa-miR-1306-5p			
435	USP15	0.216	0.390	0.443	2	hsa-miR-132-3p	hsa-miR-29a-3p			
436	HIC2	0.225	0.392	0.455	2	hsa-let-7e-5p/hsa-let-7f-5p	hsa-miR-132-3p			
437	PLEKHA3	0.225	0.392	0.455	2	hsa-let-7e-5p/hsa-let-7f-5p	hsa-miR-29a-3p			
438	SESTD1	0.225	0.392	0.455	2	hsa-let-7e-5p/hsa-let-7f-5p	hsa-miR-29a-3p			
439	AFF4	0.234	0.399	0.466	2	hsa-miR-132-3p	hsa-miR-29a-3p			

440	ANKRD52	0.233	0.399	0.583	3	hsa-let-7e-5p/hsa-let-7f-5p	hsa-miR-29a-3p	hsa-miR-378f		
441	BCL11B	0.234	0.399	0.466	2	hsa-miR-1306-5p	hsa-miR-29a-3p			
442	CHD7	0.234	0.399	0.466	2	hsa-let-7e-5p/hsa-let-7f-5p	hsa-miR-1306-5p			
443	FBXO28	0.234	0.399	0.466	2	hsa-miR-132-3p	hsa-miR-29a-3p			
444	FZD4	0.234	0.399	0.466	2	hsa-let-7e-5p/hsa-let-7f-5p	hsa-miR-29a-3p			
445	G3BP1	0.234	0.399	0.466	2	hsa-let-7e-5p/hsa-let-7f-5p	hsa-miR-29a-3p			
446	IGF2BP1	0.234	0.399	0.466	2	hsa-let-7e-5p/hsa-let-7f-5p	hsa-miR-29a-3p			
447	RIMKLA	0.234	0.399	0.466	2	hsa-let-7e-5p/hsa-let-7f-5p	hsa-miR-1306-5p			
448	SAMD12	0.234	0.399	0.466	2	hsa-let-7e-5p/hsa-let-7f-5p	hsa-miR-132-3p			
449	BRWD3	0.243	0.401	0.477	2	hsa-let-7e-5p/hsa-let-7f-5p	hsa-miR-29a-3p			
450	CBL	0.243	0.401	0.477	2	hsa-let-7e-5p/hsa-let-7f-5p	hsa-miR-378f			
451	CDK6	0.243	0.401	0.477	2	hsa-let-7e-5p/hsa-let-7f-5p	hsa-miR-29a-3p			
452	CNNM2	0.243	0.401	0.477	2	hsa-let-7e-5p/hsa-let-7f-5p	hsa-miR-29a-3p			
453	DYRK1A	0.243	0.401	0.477	2	hsa-let-7e-5p/hsa-let-7f-5p	hsa-miR-378f			
454	GRIN2B	0.243	0.401	0.477	2	hsa-let-7e-5p/hsa-let-7f-5p	hsa-miR-29a-3p			
455	KMT2D	0.243	0.401	0.477	2	hsa-let-7e-5p/hsa-let-7f-5p	hsa-miR-1306-5p			
456	CCND2	0.252	0.410	0.489	2	hsa-let-7e-5p/hsa-let-7f-5p	hsa-miR-29a-3p			
457	KDM5A	0.252	0.410	0.489	2	hsa-miR-132-3p	hsa-miR-29a-3p			
458	PLXNC1	0.262	0.412	0.500	2	hsa-let-7e-5p/hsa-let-7f-5p	hsa-miR-29a-3p			
459	SYNCRIP	0.262	0.412	0.500	2	hsa-let-7e-5p/hsa-let-7f-5p	hsa-miR-29a-3p			
460	ZBTB34	0.262	0.412	0.500	2	hsa-miR-132-3p	hsa-miR-29a-3p			

461	ANKFY1	0.281	0.421	0.523	2	hsa-let-7e-5p/hsa-let-7f-5p	hsa-miR-29a-3p			
462	CPEB3	0.281	0.421	0.523	2	hsa-let-7e-5p/hsa-let-7f-5p	hsa-miR-29a-3p			
463	CCNT2	0.290	0.428	0.534	2	hsa-let-7e-5p/hsa-let-7f-5p	hsa-miR-29a-3p			
464	CLOCK	0.300	0.428	0.545	2	hsa-let-7e-5p/hsa-let-7f-5p	hsa-miR-132-3p			
465	FIGN	0.300	0.428	0.545	2	hsa-let-7e-5p/hsa-let-7f-5p	hsa-miR-29a-3p			
466	MAPK1	0.290	0.428	0.534	2	hsa-miR-132-3p	hsa-miR-378f			
467	ATXN1	0.319	0.438	0.568	2	hsa-miR-132-3p	hsa-miR-29a-3p			
468	FRS2	0.319	0.438	0.568	2	hsa-let-7e-5p/hsa-let-7f-5p	hsa-miR-29a-3p			
469	MMP16	0.319	0.438	0.568	2	hsa-miR-132-3p	hsa-miR-29a-3p			
470	DYRK2	0.329	0.444	0.580	2	hsa-let-7e-5p/hsa-let-7f-5p	hsa-miR-132-3p			
471	MAP3K2	0.329	0.444	0.580	2	hsa-let-7e-5p/hsa-let-7f-5p	hsa-miR-29a-3p			
472	RPS6KA3	0.329	0.444	0.580	2	hsa-let-7e-5p/hsa-let-7f-5p	hsa-miR-29a-3p			
473	SCN1A	0.329	0.444	0.580	2	hsa-miR-1306-5p	hsa-miR-132-3p			
474	FOXP2	0.338	0.450	0.591	2	hsa-let-7e-5p/hsa-let-7f-5p	hsa-miR-132-3p			
475	NOVA1	0.338	0.450	0.591	2	hsa-let-7e-5p/hsa-let-7f-5p	hsa-miR-132-3p			
476	GTDC1	0.348	0.453	0.602	2	hsa-miR-132-3p	hsa-miR-29a-3p			
477	TCF4	0.348	0.453	0.602	2	hsa-miR-1306-5p	hsa-miR-29a-3p			
478	PTEN	0.358	0.463	0.614	2	hsa-miR-132-3p	hsa-miR-29a-3p			
479	SESN3	0.358	0.463	0.614	2	hsa-let-7e-5p/hsa-let-7f-5p	hsa-miR-132-3p			
480	GAN	0.367	0.466	0.625	2	hsa-let-7e-5p/hsa-let-7f-5p	hsa-miR-29a-3p			
481	KPNA4	0.377	0.472	0.636	2	hsa-let-7e-5p/hsa-let-7f-5p	hsa-miR-29a-3p			

482	NFAT5	0.387	0.478	0.648	2	hsa-miR-132-3p	hsa-miR-29a-3p			
483	HIPK2	0.396	0.481	0.659	2	hsa-let-7e-5p/hsa-let-7f-5p	hsa-miR-1306-5p			
484	KCMF1	0.396	0.481	0.659	2	hsa-let-7e-5p/hsa-let-7f-5p	hsa-miR-132-3p			
485	STRN	0.396	0.481	0.659	2	hsa-let-7e-5p/hsa-let-7f-5p	hsa-miR-29a-3p			
486	ABI2	0.406	0.491	0.670	2	hsa-miR-1306-5p	hsa-miR-378f			
487	ACVR2B	0.415	0.493	0.682	2	hsa-let-7e-5p/hsa-let-7f-5p	hsa-miR-132-3p			
488	BNC2	0.415	0.493	0.682	2	hsa-let-7e-5p/hsa-let-7f-5p	hsa-miR-132-3p			
489	ZBTB10	0.415	0.493	0.682	2	hsa-let-7e-5p/hsa-let-7f-5p	hsa-miR-29a-3p			
490	ONECUT2	0.435	0.507	0.705	2	hsa-let-7e-5p/hsa-let-7f-5p	hsa-miR-29a-3p			
491	QKI	0.435	0.507	0.705	2	hsa-miR-132-3p	hsa-miR-29a-3p			
492	UBN2	0.435	0.507	0.705	2	hsa-let-7e-5p/hsa-let-7f-5p	hsa-miR-29a-3p			
493	CELF2	0.444	0.511	0.716	2	hsa-miR-132-3p	hsa-miR-29a-3p			
494	NFIA	0.444	0.511	0.716	2	hsa-miR-132-3p	hsa-miR-29a-3p			
495	AGO3	0.454	0.514	0.727	2	hsa-let-7e-5p/hsa-let-7f-5p	hsa-miR-29a-3p			
496	ZBTB20	0.457	0.514	0.795	3	hsa-miR-132-3p	hsa-miR-29a-3p	hsa-miR-378f		
497	AAK1	0.482	0.533	0.761	2	hsa-let-7e-5p/hsa-let-7f-5p	hsa-miR-378f			
498	NFIB	0.556	0.586	0.852	2	hsa-miR-132-3p	hsa-miR-29a-3p			
499	NUFIP2	0.556	0.586	0.852	2	hsa-miR-29a-3p	hsa-miR-378f			

Abbreviation: FDR, false discovery rate.

Table S6. KEGG analysis using 499 putative target transcripts.

Term name		Term id	Adjusted p value	Gene counts	Associated genes
1	Hepatocellular carcinoma	KEGG:05225	1.54E-06	19	PIK3CA,GSK3B,MAPK1,IGF1R,AKT3,PTEN,CDKN1A,NRAS,FZD5,APC,APC2,RB1, ARID1B,WNT9B,SMARCC1,GAB1,SMAD2,FZD4,CDK6
2	Focal adhesion	KEGG:04510	1.54E-06	21	PIK3CA,GSK3B,MAPK1,MAPK8,CRK,COL4A2,PDGFB,COL4A1,COL4A6,IGF1, COL4A4,IGF1R,AKT3,PTEN,COL1A2,COL1A1,THBS1,CCND2,COL4A5,DIAPH1,COL9A1
3	FoxO signaling pathway	KEGG:04068	4.17E-06	16	PIK3CA,MAPK1,PRKAA2,FOXO3,MAPK8,PRKAB2,IGF1,IGF1R,AKT3,PTEN,CDKN1A, NRAS,CCND2,STAT3,SIRT1,NLK
4	EGFR tyrosine kinase inhibitor resistance	KEGG:01521	1.00E-05	12	PIK3CA,GSK3B,MAPK1,FOXO3,PDGFB,IGF1,IGF1R,AKT3,PTEN,NRAS,STAT3,GAB1
5	PI3K-Akt signaling pathway	KEGG:04151	2.80E-05	25	PIK3CA,GSK3B,MAPK1,PRKAA2,FOXO3,COL4A2,PDGFB,COL4A1,COL4A6,IGF1, COL4A4,IGF1R,AKT3,PTEN,CDKN1A,COL1A2,COL1A1,THBS1,CREB5,GHR,NRAS, CCND2,COL4A5,COL9A1,CDK6
6	ErbB signaling pathway	KEGG:04012	7.73E-05	11	PIK3CA,GSK3B,MAPK1,MAPK8,CRK,CBL,AKT3,CDKN1A,NRAS,HBEGF,GAB1
7	Prolactin signaling pathway	KEGG:04917	8.87E-05	10	PIK3CA,GSK3B,MAPK1,FOXO3,MAPK8,AKT3,NRAS,CCND2,STAT3,SOCS7
8	Insulin resistance	KEGG:04931	1.44E-04	12	PIK3CA,GSK3B,PRKAA2,MAPK8,PRKAB2,RPS6KA3,AKT3,PTEN,PPARGC1A,CREB5, STAT3,MLXIP
9	Endocrine resistance	KEGG:01522	1.92E-04	11	PIK3CA,MAPK1,MAPK8,IGF1,IGF1R,AKT3,CDKN1A,NRAS,HBEGF,RB1,NCOR1
10	mTOR signaling pathway	KEGG:04150	2.35E-04	14	PIK3CA,GSK3B,MAPK1,PRKAA2,RPS6KA3,IGF1,IGF1R,AKT3,PTEN,NRAS,FZD5, WNT9B, RNF152,FZD4
11	Wnt signaling pathway	KEGG:04310	4.78E-04	14	GSK3B,MAPK8,CCND2,CHD8,FZD5,APC,APC2,PRICKLE2,WNT9B,PPP3CA,DAAM1, VANGL1, NLK,FZD4
12	PD-L1 expression and PD-1 checkpoint pathway in cancer	KEGG:05235	4.78E-04	10	PIK3CA,MAPK1,MAP3K3,MAP2K6,AKT3,PTEN,NRAS,RASGRP1,STAT3,PPP3CA
13	MAPK signaling pathway	KEGG:04010	2.19E-03	18	MAPK1,MAPK8,MAP4K4,MAP3K3,MAP2K6,MAP3K2,RPS6KA3,CRK,PDGFB,IGF1, IGF1R,AKT3,NRAS,DUSP9,CACNG4,RASGRP1,PPP3CA,NLK
14	Hippo signaling pathway	KEGG:04390	2.89E-03	12	GSK3B,CCND2,AMOT,MOB1B,FZD5,APC,APC2,WNT9B,BMP2,SMAD2,BMPR1A,FZD4
15	Insulin signaling pathway	KEGG:04910	3.18E-03	11	PIK3CA,GSK3B,MAPK1,PRKAA2,MAPK8,PRKAB2,CRK,CBL,AKT3,PPARGC1A,NRAS
16	Alcoholic liver disease	KEGG:04936	4.03E-03	11	GSK3B,PRKAA2,FOXO3,MAPK8,PRKAB2,MAP2K6,AKT3,PPARGC1A,CAMKK2, SIRT1,TRAF3
17	Adipocytokine signaling pathway	KEGG:04920	7.11E-03	7	PRKAA2,MAPK8,PRKAB2,AKT3,PPARGC1A,STAT3,CAMKK2
18	TGF-beta signaling pathway	KEGG:04350	9.32E-03	8	MAPK1,THBS1,E2F5,BMP2,ACVR2A,SMAD2,BMPR1A,ACVR2B

19	p53 signaling pathway	KEGG:04115	9.32E-03	7	SESN3,IGF1,PTEN,CDKN1A,THBS1,CCND2,CDK6

Figure S1. Summary of study design.

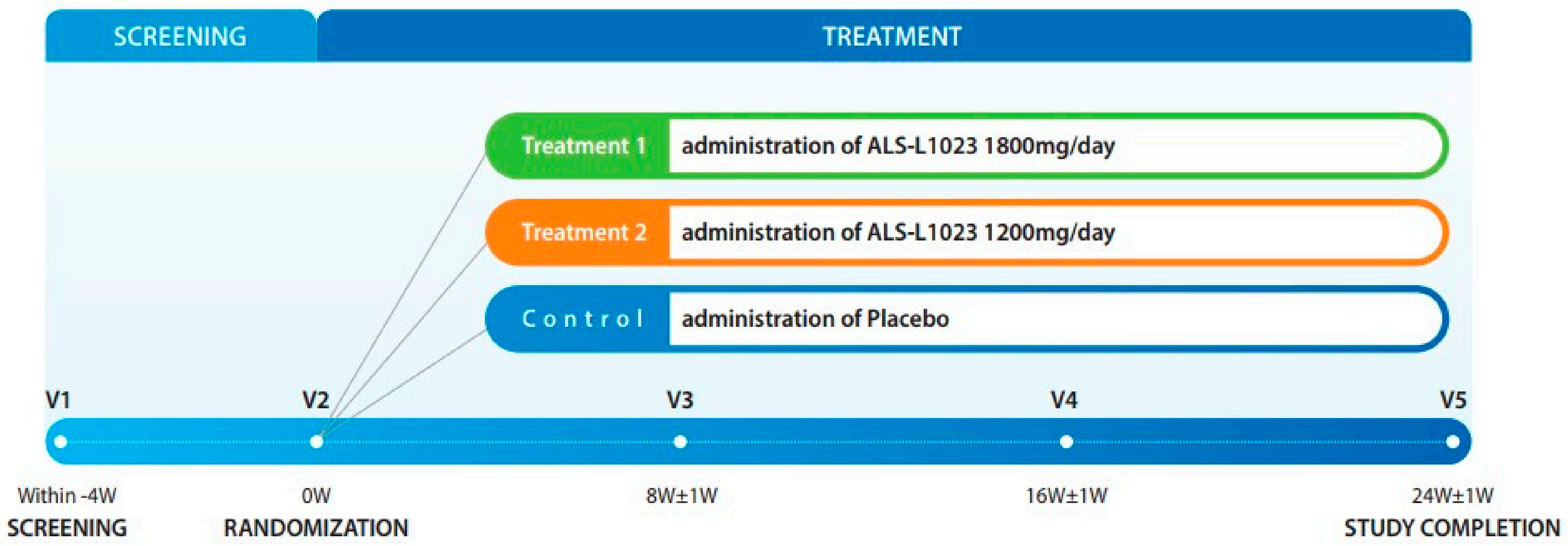

Figure S2. MRI-PDFF change in the PPS

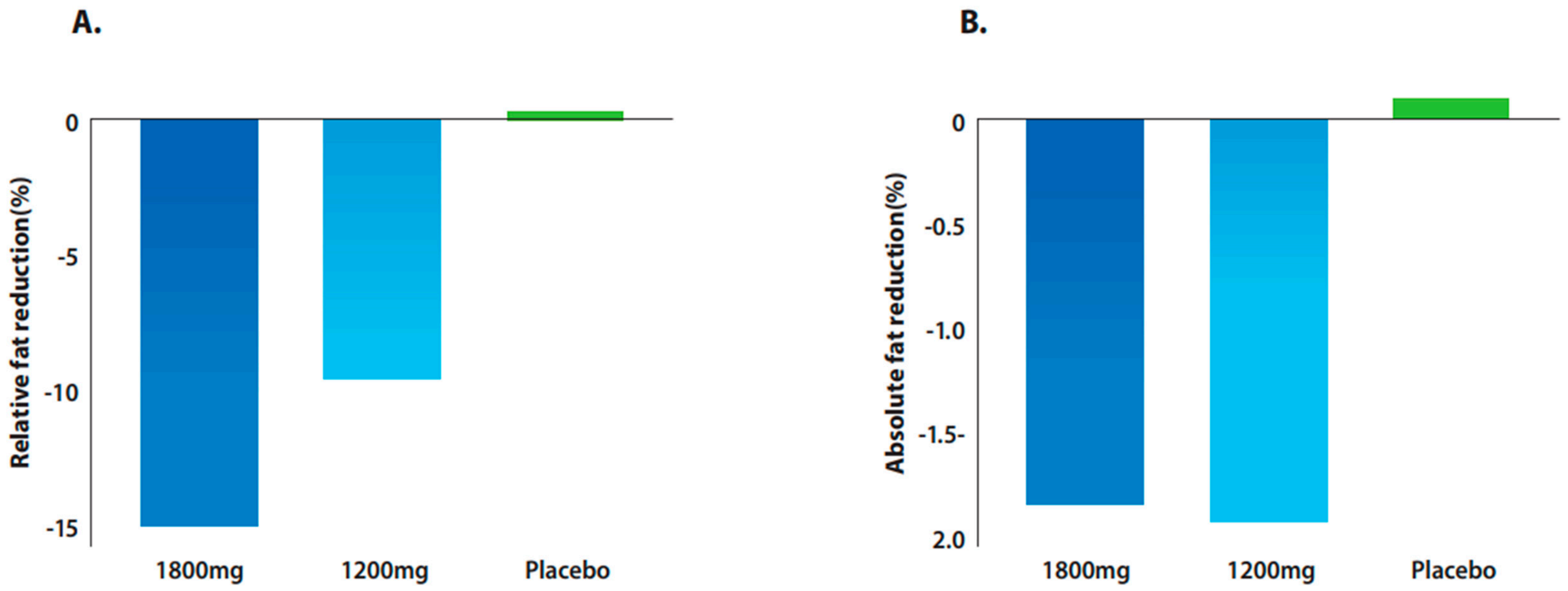

Figure S3. Change of liver biochemistry in the PPS

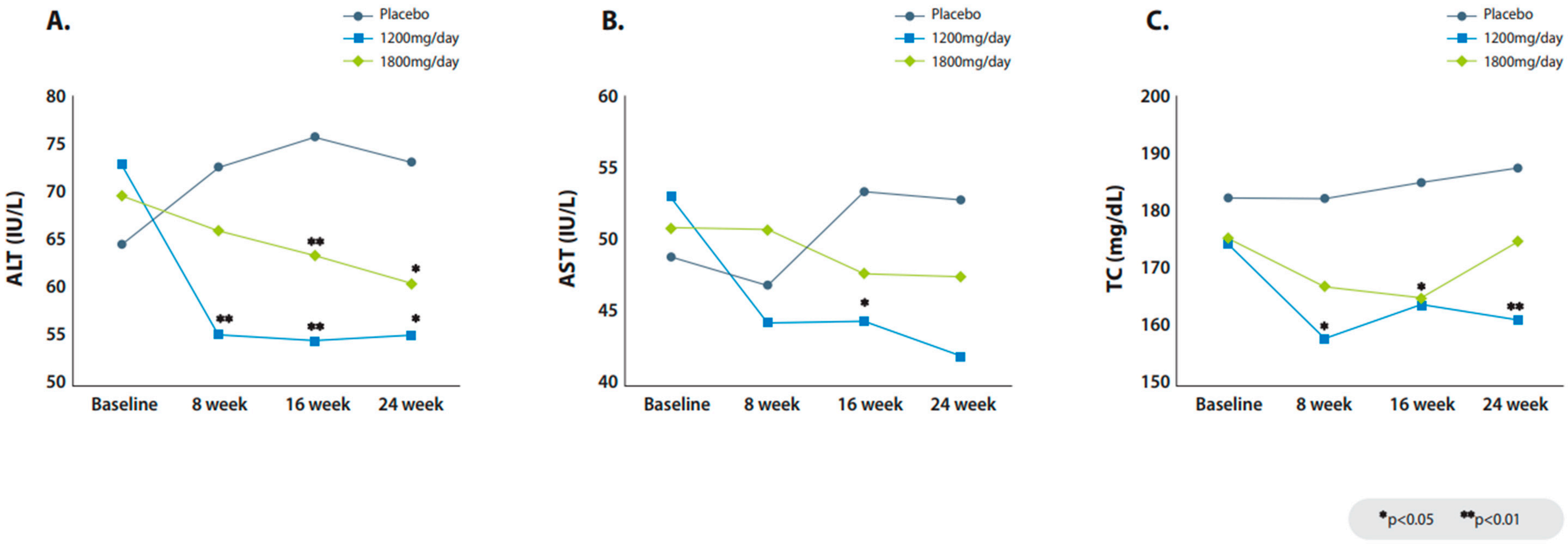

Supplement: Supplementary file 1 [file pharmaceuticals-16-00623-s001.zip › pharmaceuticals-2342602-supplementary.pdf]
